# Comparative proteomic analysis of human lung telocytes with fibroblasts

**DOI:** 10.1111/jcmm.12290

**Published:** 2014-03-28

**Authors:** Yonghua Zheng, Dragos Cretoiu, Guoquan Yan, Sanda Maria Cretoiu, Laurentiu M Popescu, Xiangdong Wang

**Affiliations:** a Department of Respirology, Xinhua Hospital Affiliated to Shanghai Jiao Tong University School of MedicineShanghai, China; b Division of Cellular and Molecular Medicine, Carol Davila University of Medicine and PharmacyBucharest, Romania; c Department of Molecular Medicine, Victor Babeş National Institute of PathologyBucharest, Romania; d Department of Chemistry, Institute of Biomedical Sciences, Fudan UniversityShanghai, China; e Department of Ultrastructural Pathology, Victor Babeş National Institute of PathologyBucharest, Romania; f Division of Advanced Studies, Victor Babeş National Institute of PathologyBucharest, Romania; g Department of Pulmonary Medicine and Biomedical Research Center, Zhongshan Hospital, Fudan UniversityShanghai, China

**Keywords:** proteomics, telocytes, fibroblasts, lung, isobaric tags for relative and absolute quantification (iTRAQ), LC-MS/MS

## Abstract

Telocytes (TCs) were recently described as interstitial cells with very long prolongations named telopodes (Tps; http://www.telocytes.com). Establishing the TC proteome is a priority to show that TCs are a distinct type of cells. Therefore, we examined the molecular aspects of lung TCs by comparison with fibroblasts (FBs). Proteins extracted from primary cultures of these cells were analysed by automated 2-dimensional nano-electrospray ionization liquid chromatography tandem mass spectrometry (2D Nano-ESI LC-MS/MS). Differentially expressed proteins were screened by two-sample *t*-test (*P* < 0.05) and fold change (>2), based on the bioinformatics analysis. We identified hundreds of proteins up- or down-regulated, respectively, in TCs as compared with FBs. TC proteins with known identities are localized in the cytoskeleton (87%) and plasma membrane (13%), while FB up-regulated proteins are in the cytoskeleton (75%) and destined to extracellular matrix (25%). These identified proteins were classified into different categories based on their molecular functions and biological processes. While the proteins identified in TCs are mainly involved in catalytic activity (43%) and as structural molecular activity (25%), the proteins in FBs are involved in catalytic activity (24%) and in structural molecular activity, particularly synthesis of collagen and other extracellular matrix components (25%). Anyway, our data show that TCs are completely different from FBs. In conclusion, we report here the first extensive identification of proteins from TCs using a quantitative proteomics approach. Protein expression profile shows many up-regulated proteins *e.g*. myosin-14, periplakin, suggesting that TCs might play specific roles in mechanical sensing and mechanochemical conversion task, tissue homoeostasis and remodelling/renewal. Furthermore, up-regulated proteins matching those found in extracellular vesicles emphasize TCs roles in intercellular signalling and stem cell niche modulation. The novel proteins identified in TCs will be an important resource for further proteomic research and it will possibly allow biomarker identification for TCs. It also creates the premises for understanding the pathogenesis of some lung diseases involving TCs.

## Introduction

Telocytes (TCs) are a novel defined cell type 1, resident in the stromal space of several mammalian and human organs 2–21, including in human lungs 22–24 (visit http://www.telocytes.com). TCs are characterized by very long and thin prolongations called telopodes (Tps) which suddenly emerge from a small cell body. Telopodes are tens to hundreds of micrometres long and have many dilations (podoms) interconnected by thin regions (podomeres) throughout their length 16. The TCs interconnect with each other through point cell-to-cell contacts or gap junctions, forming a three-dimensional network 25,26 and set up close contacts with other interstitial cells (*e.g*. macrophages, mast cells, lymphocytes) 12,27. Frequently, TCs could be observed in close vicinity with nerve cells, blood capillaries and stem cell niches 28,29. Although the specific function(s) of TCs are still not established, it became increasingly clear that they have an integrative role, possibly for stem cells 7,25,30,31, involving a long-distance communication confirmed by the presence of exosomes/ectosomes 16.

Telocytes were characterized ultrastructurally, immunophenotypically, electrophysiologically and their gene and microRNA profile were analysed 16,31–35. Telocytes are also involved in pathologies 5,29,36–41.

However, the protein expression profile for this type of cells has not been reported yet. In a previous study, we reported the gene expression profile of mice lung TCs compared to fibroblasts (FBs), and the results showed that more than ˜1000 genes were found up- or down-regulated respectively 42. Significantly improved technology in quantitative MS-based proteomics permits the measurement of relative protein loads in cell culture or tissue samples with unique precision 43,44.

To prevent further confusion between TCs and other interstitial (stromal) cells, particularly with FBs and the so-called FBs-like cells, we carried out a comprehensive study using iTRAQ-coupled 2D LC-MS/MS analysis to identify and quantify the proteins. This study is the first that allows major insight into proteome differences between these cells in human lung and which identify the proteins that are specifically expressed in TCs.

## Material and methods

### Cell lines and tissue sampling

Human lung samples were obtained from the patients undergoing surgery for lung cancer. The normal tissue was defined as being located at a distance of at least 15 cm from the tumour tissue and verified by light microscopy. The application of human tissue for research was approved by the Ethical Evaluation Committee of Zhongshan Hospital, Fudan University, Shanghai, China. Human lung fibroblast cell lines were obtained from Chinese Academy of Science (Cat. no. GNHu28; Shanghai, China).

### Primary cell culture and lysis of lung TCs

The methods of isolation and culture of lung TCs were previously described by Zheng *et al*. 22. Briefly, lung tissue was cut into small pieces and harvested under sterile conditions and collected into sterile tubes containing DMEM (Gibco, Grand Island, NY, USA), supplemented with 100 UI/ml penicillin and 0.1 mg/ml streptomycin (Sigma Chemical, St. Louis, MO, USA), and the samples were brought to the cell culture room immediately. Samples were further rinsed with sterile DMEM and minced into fragments about 1 mm^3^, which were then incubated at 37°C for 4 hrs on an orbital shaker, with 1 mg/ml type II collagenase (Sigma-Aldrich, St. Louis, MO, USA) in PBS without Ca^2+^ and Mg^2+^. Dispersed cells were separated from non-digested tissue by the filtration through a 40-μm diameter cell strainer (BD Falcon, Franklin, NJ, USA), harvested by centrifugation and resuspended in DMEM supplemented with 10% foetal calf serum (Gibco), 100 UI/ml penicillin and 0.1 mg/ml streptomycin. Cell density was counted in a haemocytometer and viability was assessed using the Trypan blue. Cells were distributed in 25 cm^2^ culture flasks at a density of 1 × 10^5^ cells/cm^2^ and maintained at 37°C in a humidified atmosphere (5% CO_2_) until becoming semiconfluent (usually 4 days after plating). Culture medium was changed every 48 hrs. Cultured cells were examined by phase contrast microscope, under an inverted Olympus phase contrast microscope (1 × 51). Cells (1 × 10^5^) were placed in 10-cm dishes with 10 ml high glucose DMEM (Gibco) complete medium, including 10% foetal calf serum (Gibco), 100 UI/ml penicillin and 0.1 mg/ml streptomycin (Sigma Chemical) in a humidified atmosphere of 5% CO_2_ at 37°C. Confluent cells were trypsinized at day 5 and day 10 respectively. Approximately 10^6^ cells from day 5 or day 10 were resuspended in a solution of 9.5 moles/litre urea, 1% dithiothreitol (DTT), 40 mg/ml protease inhibitor cocktail, 0.2 mmoles/litre Na_2_VO_3_ and 1 mmole/litre NaF. The mixture was incubated and stirred by end-over-end rotation at 4°C for 60 min. The resultant suspension was centrifuged at 40,000 × g for 1 hr at 15°C. The supernatant was stored in small aliquots at −80°C, and the protein concentration was determined using a modified Bradford method.

### Automated 2-D nano-ESI LC-MS/MS analysis of peptides

Proteins extracted from primary cultures of TCs and FBs were analysed by automated 2-dimensional nano-electrospray ionization liquid chromatography tandem mass spectrometry as was previously described by Wang *et al*. 45 and Jin *et al*.^46^.

### Sample preparation

The samples were ground in liquid nitrogen. One millilitre of lysis buffer (8 M urea, 1× Protease Inhibitor Cocktail; Roche Ltd., Basel, Switzerland) was added to sample, followed by sonication on ice and centrifugation at 29,000 × g. for 10 min. at 4°C. The supernatant was transferred to a fresh tube and stored at −80°C until needed.

### iTRAQ labelling and protein digestion

For each sample, proteins were precipitated with ice-cold acetone and then were redissolved in the dissolution buffer (0.5 M triethylammonium bicarbonate, 0.1% SDS). Then proteins were quantified by the bicinchoninic acid protein assay, and 100 μg of protein was tryptically digested and the resultant peptide mixture was labelled using chemicals from the iTRAQ reagent kit (Applied Biosystems, Foster City, CA, USA). Disulphide bonds were reduced in 5 mM Tris-(2-carboxyethy) phosphine (TCEP) for 1 hr at 60°C, followed by blocking cysteine residues in 10 mM methyl methanethiosulfonate (MMTS) for 30 min. at room temperature, before digestion with sequence-grade modified trypsin (Promega, Madison, WI, USA). For labelling, each iTRAQ reagent was dissolved in 50 μl of isopropanol and added to the respective peptide mixture.

Proteins were labelled with the iTRAQ tags as follows: Fibroblast (5 days) – 114 isobaric tag, TCs (5 days) – 116 isobaric tag, Fibroblast (10 days) – 118 isobaric tag, TCs (10 days) – 121 isobaric tag. The labelled samples were combined and dried in vacuo. A SepPac™ C18 cartridge (1 cm^3^/50 mg, Waters Corporation, Milford, MA, USA) was used to remove the salt buffer and then was dried in a vacuum concentrator for the next step.

### High pH reverse phase separation

The peptide mixture was redissolved in the buffer A (buffer A: 20 mM ammonium formate in water, pH 10.0, adjusted with ammonium hydroxide), and then fractionated by high pH separation using a Aquity UPLC system (Waters Corporation) connected to a reverse phase column (XBridge C18 column, 2.1 × 150 mm, 3.5 μm, 300 Å, Waters Corporation). High pH separation was performed with a linear gradient. Starting from 2% B to 40% B in 45 min. (B: 20 mM ammonium formate in 90% ACN, pH 10.0, adjusted with ammonium hydroxide). The column was re-equilibrated at initial conditions for 15 min. The column flow rate was maintained at 200 μl/min. and column temperature was maintained at room temperature 47. Fourteen fractions were collected, and each fraction was dried in a vacuum concentrator for the next step.

### Low pH nano-HPLC-MS/MS analysis

The peptides were resuspended with 80 μl solvent C (C: water with 0.1% formic acid; D: ACN with 0.1% formic acid), separated by nanoLC and analysed by on-line electrospray tandem mass spectrometry. The experiments were performed on a Nano Aquity UPLC system (Waters Corporation) connected to an LTQ Orbitrap XL mass spectrometer (Thermo Electron Corp., Bremen, Germany) equipped with an online nanoelectrospray ion source (Michrom Bioresources, Auburn, CA, USA). 20 μl peptide sample was loaded onto the Thermo Scientific Acclaim PepMap C18 column (100 μm × 2 cm, 3 μm particle size), with a flow of 10 μl/min. for 5 min. and subsequently separated on the analytical column (Acclaim PepMap C18, 75 μm × 15 cm) with a linear gradient, from 5% D to 45% D in 165 min. The column was re-equilibrated at initial conditions for 15 min. The column flow rate was maintained at 300 nl/min. and column temperature was maintained at 35°C. The electrospray voltage of 1.9 kV *versus* the inlet of the mass spectrometer was used.

LTQ Orbitrap XL mass spectrometer was operated in the data-dependent mode to switch automatically between MS and MS/MS acquisition. Survey full-scan MS spectra (m/z 400-1600) were acquired in the Orbitrap with a mass resolution of 30,000 at m/z 400, followed by five sequential HCD-MS/MS scans. The automatic gain control was set to 500,000 ions to prevent over-filling of the ion trap. The minimum MS signal for triggering MS/MS was set to 1000. In all cases, one microscan was recorded. MS/MS scans were acquired in the Orbitrap with a mass resolution of 7500. The dissociation mode was HCD (higher energy C-trap dissociation). Dynamic exclusion was used with two repeat counts, 10-sec. repeat duration, and the m/z values triggering MS/MS were put on an exclusion list for 120 sec. For MS/MS, precursor ions were activated using 40% normalized collision energy and an activation time of 30 ms.

### Database searching, criteria and protein parameterization

Protein identification and quantification for the iTRAQ experiment was performed with the ProteinPilot software version 4.0 (Applied Biosystems). The database was the Human UniProtKB/Swiss-Prot database (Release 2011_10_15, with 20248 sequences). The Paragon Algorithm in ProteinPilot software was used for peptide identification and isoform-specific quantification. The data search parameters were set up as follows: trypsin (KR) cleavage with two missed cleavage was considered; fixed modification in cysteines by MMTS; iTRAQ modification in peptide N termini, methionine oxidation and iTRAQ modification in lysine residues were set as variable modification. To minimize false positive results, a strict cut-off for protein identification was applied with the unused ProtScore ≥1.3, which corresponds to a confidence limit of 95%, and at least two peptides with the 95% confidence were considered for protein quantification. The resulting data set was auto bias corrected to get rid of any variations imparted because of unequal mixing during combining different labelled samples. For iTRAQ quantification, the peptide for quantification was automatically selected by Pro Group algorithm (at least two peptides with 99% confidence) to calculate the reporter peak area, error factor and p-value. For the selection of differentially expressed proteins, we considered the following situation: (*i*) the proteins must contain at least two unique high-scoring peptides; (*ii*) the proteins must have a *P* < 0.05 and the proteins identified with mass tag changes ratio must be ≥1.3 or ≤0.75.

Differentially expressed proteins were screened by two-sample *t*-test (*P* < 0.05) and fold change (>2), based on the bioinformatics analysis.

### Data set analysis

MS/MS fragmentation spectra were analysed using PEAKS search engine tool (PEAKS Studio 7; Bioinformatics Solutions Inc., Waterloo, ON, Canada).

The protein details obtained were analysed for the function, process, location, by PANTHER (Protein ANalysis THrough Evolutionary Relationships) classification system (http://www.pantherdb.org/) 48 which is based on a controlled dialect to describe a protein regarding its subcellular localization, molecular function or biological process.

Top over-expressed proteins were used to create radar (spider) plots Using Microsoft Excel.

## Results

**Figure 1:**
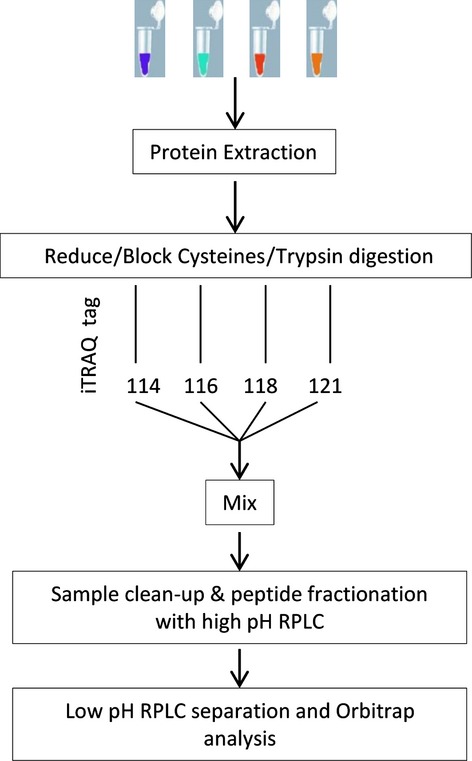
Proteomic process flow chart illustrating the steps involved in the differential analysis of TCs and FBs proteome in cell culture

This study utilizes a novel proteomic approach based on isobaric tags for relative and absolute quantification (iTRAQ) using nano liquid chromatography tandem mass spectrometry analysis to identify specifically over expressed proteins in TCs comparative to FBs. Figure 1 shows the sequence of connected procedural steps used in this protocol.

We identified a total of 1609 proteins by iTRAQ technology using nano LC-MS/MS analysis. The temporal proteome was evaluated at 5 and 10 days, respectively, and the selected lists of differentially up-regulated proteins identified in TCs and FBs, in cell culture, are shown in Table 1 (5th day) and Table 2 (10th day).

**Table 1:** Selected list of top 56 proteins identified with more than twofold change in TCs *versus*FBs at 5th day sorted by iTRAQ ratio and presenting the number of peptides hits

Accession	Protein name	Peptides (95%)	%Cov (95)	iTRAQ ratio FBs:TCs	Fold enrichment in TCs	*P*
MYH14_HUMAN	Myosin-14	18	7.67	0.064	15.72	0.025
SODM_HUMAN	Superoxide dismutase [Mn], mitochondrial	10	35.14	0.104	9.6	0.000
ALDH2_HUMAN	Aldehyde dehydrogenase, mitochondrial	5	10.06	0.164	6.1	0.000
PTGIS_HUMAN	Prostacyclin synthase	7	15.80	0.199	5.03	0.005
APOH_HUMAN	Beta-2-glycoprotein 1	2	4.35	0.210	4.75	0.041
PPBT_HUMAN	Alkaline phosphatase, tissue-non-specific isozyme	2	4.01	0.260	3.85	0.043
PEPL_HUMAN	Periplakin	4	2.85	0.284	3.52	0.032
AL1B1_HUMAN	Aldehyde dehydrogenase X, mitochondrial	5	13.93	0.313	3.2	0.024
THIM_HUMAN	3-ketoacyl-CoA thiolase, mitochondrial	12	33.50	0.329	3.04	0.036
KAD2_HUMAN	Adenylate kinase 2, mitochondrial	8	41.42	0.336	2.97	0.001
PLOD2_HUMAN	Procollagen-lysine, 2-oxoglutarate 5-dioxygenase 2	15	22.39	0.367	2.72	0.000
AT1A1_HUMAN	Sodium/potassium-transporting ATPase subunit α-1	17	18.77	0.375	2.67	0.000
COX5B_HUMAN	Cytochrome c oxidase subunit 5B, mitochondrial	4	34.11	0.380	2.63	0.006
DNJC3_HUMAN	DnaJ homologue subfamily C member 3	2	3.77	0.392	2.55	0.024
PRDX3_HUMAN	Thioredoxin-dependent peroxide reductase, mitochondrial	6	23.83	0.395	2.53	0.028
SUCA_HUMAN	Succinyl-CoA ligase [GDP-forming] subunit α, mitochondrial	5	17.63	0.399	2.51	0.008
COX5A_HUMAN	Cytochrome c oxidase subunit 5A, mitochondrial	6	58.67	0.408	2.45	0.000
CH10_HUMAN	10 kD heat shock protein, mitochondrial	12	73.53	0.409	2.44	0.003
CH60_HUMAN	60 kD heat shock protein, mitochondrial	60	62.30	0.414	2.42	0.003
SQRD_HUMAN	Sulphide:quinone oxidoreductase, mitochondrial	9	19.78	0.421	2.38	0.000
ERP29_HUMAN	Endoplasmic reticulum resident protein 29	7	28.74	0.422	2.37	0.001
K2C1_HUMAN	Keratin, type II cytoskeletal 1	12	15.06	0.435	2.3	0.004
ERP44_HUMAN	Endoplasmic reticulum resident protein 44	4	8.13	0.442	2.26	0.002
CATA_HUMAN	Catalase	5	12.90	0.460	2.18	0.007
ETFB_HUMAN	Electron transfer flavoprotein subunit β	6	25.88	0.473	2.11	0.008
SSBP_HUMAN	Single-stranded DNA-binding protein, mitochondrial	6	50.68	0.476	2.1	0.028
NLTP_HUMAN	Non-specific lipid-transfer protein	9	12.43	0.477	2.1	0.010
PDIA3_HUMAN	Protein disulphide-isomerase A3	45	52.48	0.480	2.08	0.019
IDHP_HUMAN	Isocitrate dehydrogenase [NADP], mitochondrial	5	9.29	0.492	2.03	0.000
SPTB2_HUMAN	Spectrin β chain, brain 1	43	19.42	0.493	2.03	0.000
RT36_HUMAN	28S ribosomal protein S36, mitochondrial	2	30.10	0.494	2.02	0.036
THIO_HUMAN	Thioredoxin	5	51.43	2.006	2.01	0.008
KPYM_HUMAN	Pyruvate kinase isozymes M1/M2	36	67.23	2.035	2.03	0.016
CD166_HUMAN	CD166 antigen	4	11.32	2.055	2.06	0.006
WDR1_HUMAN	WD repeat-containing protein 1	10	18.15	2.061	2.06	0.001
RS2_HUMAN	40S ribosomal protein S2	10	31.06	2.088	2.09	0.000
ACLY_HUMAN	ATP-citrate synthase	11	11.08	2.089	2.09	0.000
FLNC_HUMAN	Filamin-C	73	32.40	2.160	2.16	0.000
GSTO1_HUMAN	Glutathione S-transferase omega-1	2	9.54	2.181	2.18	0.028
VAT1_HUMAN	Synaptic vesicle membrane protein VAT-1 homologue	12	31.81	2.183	2.18	0.015
CRTAP_HUMAN	Cartilage-associated protein	2	3.74	2.273	2.27	0.049
SYVC_HUMAN	Valyl-tRNA synthetase	3	2.37	2.276	2.28	0.010
VIME_HUMAN	Vimentin	200	83.69	2.603	2.6	0.000
5NTD_HUMAN	5′-nucleotidase	6	14.81	2.603	2.6	0.000
NEST_HUMAN	Nestin	22	16.84	2.615	2.62	0.006
PLIN3_HUMAN	Perilipin-3	9	26.50	2.625	2.63	0.000
ANXA6_HUMAN	Annexin A6	21	31.35	2.714	2.71	0.005
SERA_HUMAN	D-3-phosphoglycerate dehydrogenase	4	8.07	2.773	2.77	0.017
G3P_HUMAN	Glyceraldehyde-3-phosphate dehydrogenase	42	64.48	2.819	2.82	0.001
S10AD_HUMAN	Protein S100-A13	5	44.90	2.823	2.82	0.000
RL15_HUMAN	60S ribosomal protein L15	2	7.84	3.130	3.13	0.006
LEG1_HUMAN	Galectin-1	27	91.11	3.133	3.13	0.000
GDIR1_HUMAN	Rho GDP-dissociation inhibitor 1	4	18.14	3.241	3.24	0.001
FSCN1_HUMAN	Fascin	6	16.63	3.255	3.25	0.004
SCRN1_HUMAN	Secernin-1	2	5.80	3.734	3.73	0.019
CO6A3_HUMAN	Collagen α-3(VI) chain	41	16.53	4.707	4.71	0.000

**Table 2:** Selected list of top 56 proteins identified with more than twofold change in TCs *versus*FBs at 10th day sorted by iTRAQ ratio and presenting the number of petides hits

Accession	Protein name	Peptides (95%)	%Cov (95)	iTRAQ ratio FBs:TCs	Fold enrichment in TCs	*P*
SODM_HUMAN	Superoxide dismutase [Mn], mitochondrial	10	35.14	0.120	8.36	0.001
PTGIS_HUMAN	Prostacyclin synthase	7	15.8	0.123	8.12	0.001
MYH14_HUMAN	Myosin-14	18	7.669	0.144	6.96	0.036
PLOD2_HUMAN	Procollagen-lysine,2-oxoglutarate 5-dioxygenase 2	15	22.39	0.211	4.74	0.000
ANXA3_HUMAN	Annexin A3	3	12.69	0.231	4.32	0.027
ICAM1_HUMAN	Intercellular adhesion molecule 1	6	14.66	0.267	3.75	0.001
NAMPT_HUMAN	Nicotinamide phosphoribosyltransferase	3	5.906	0.325	3.08	0.005
CYB5_HUMAN	Cytochrome b5	4	32.09	0.333	3	0.004
EZRI_HUMAN	Ezrin	19	31.4	0.352	2.84	0.002
MYH10_HUMAN	Myosin-10	45	19.59	0.419	2.38	0.007
FLNB_HUMAN	Filamin-B	96	41.78	0.430	2.33	0.000
THIM_HUMAN	3-ketoacyl-CoA thiolase, mitochondrial	12	33.5	0.434	2.3	0.000
SQRD_HUMAN	Sulphide:quinone oxidoreductase, mitochondrial	9	19.78	0.435	2.3	0.000
PLAK_HUMAN	Junction plakoglobin	3	4.564	0.447	2.24	0.011
DHB4_HUMAN	Peroxisomal multifunctional enzyme type 2	11	25	0.456	2.19	0.030
KAD2_HUMAN	Adenylate kinase 2, mitochondrial	8	41.42	0.484	2.07	0.000
TAGL_HUMAN	Transgelin	15	75.12	1.996	2	0.000
EHD2_HUMAN	EH domain-containing protein 2	8	17.13	2.021	2.02	0.000
RL18A_HUMAN	60S ribosomal protein L18a	3	17.61	2.022	2.02	0.002
RL13A_HUMAN	60S ribosomal protein L13a	2	6.897	2.023	2.02	0.003
PTRF_HUMAN	Polymerase I and transcript release factor	17	42.82	2.024	2.02	0.036
VINC_HUMAN	Vinculin	38	36.95	2.027	2.03	0.000
KPYM_HUMAN	Pyruvate kinase isozymes M1/M2	36	67.23	2.054	2.05	0.004
GSTO1_HUMAN	Glutathione S-transferase omega-1	2	9.544	2.059	2.06	0.008
LASP1_HUMAN	LIM and SH3 domain protein 1	6	22.61	2.068	2.07	0.000
THIO_HUMAN	Thioredoxin	5	51.43	2.072	2.07	0.017
CSRP1_HUMAN	Cysteine and glycine-rich protein 1	3	21.76	2.105	2.11	0.003
GDIR1_HUMAN	Rho GDP-dissociation inhibitor 1	4	18.14	2.121	2.12	0.024
CNN2_HUMAN	Calponin-2	9	37.86	2.131	2.13	0.000
SEPT9_HUMAN	Septin-9	3	5.119	2.158	2.16	0.010
PROF1_HUMAN	Profilin-1	23	75.71	2.176	2.18	0.000
CO1A2_HUMAN	Collagen α-2(I) chain	9	7.906	2.202	2.2	0.012
CD166_HUMAN	CD166 antigen	4	11.32	2.203	2.2	0.009
CD44_HUMAN	CD44 antigen	7	9.569	2.251	2.25	0.012
RL24_HUMAN	60S ribosomal protein L24	6	31.21	2.256	2.26	0.002
STMN1_HUMAN	Stathmin	6	32.21	2.303	2.3	0.003
5NTD_HUMAN	5′-nucleotidase	6	14.81	2.309	2.31	0.007
G3P_HUMAN	Glyceraldehyde-3-phosphate dehydrogenase	42	64.48	2.349	2.35	0.000
ANXA5_HUMAN	Annexin A5	30	54.37	2.409	2.41	0.003
FSCN1_HUMAN	Fascin	6	16.63	2.467	2.47	0.001
LEG1_HUMAN	Galectin-1	27	91.11	2.472	2.47	0.000
PLIN3_HUMAN	Perilipin-3	9	26.5	2.568	2.57	0.000
A2MG_HUMAN	α-2-macroglobulin	7	4.342	2.606	2.61	0.027
H15_HUMAN	Histone H1.5	7	23.45	2.692	2.69	0.009
MAP1B_HUMAN	Microtubule-associated protein 1B	11	5.146	2.700	2.7	0.036
VAT1_HUMAN	Synaptic vesicle membrane protein VAT-1 homologue	12	31.81	2.717	2.72	0.000
LEG3_HUMAN	Galectin-3	10	30.4	2.800	2.8	0.000
MFGM_HUMAN	Lactadherin	6	17.05	2.844	2.84	0.019
VIME_HUMAN	Vimentin	200	83.69	2.848	2.85	0.000
NEST_HUMAN	Nestin	22	16.84	2.865	2.87	0.007
NQO1_HUMAN NAD(P)	H dehydrogenase [quinone] 1	2	7.664	2.874	2.87	0.006
H12_HUMAN	Histone H1.2	7	30.99	3.039	3.04	0.001
CSRP2_HUMAN	Cysteine and glycine-rich protein 2	1	7.772	3.135	3.14	0.022
SH3L3_HUMAN	SH3 domain-binding glutamic acid-rich-like protein 3	3	34.41	3.252	3.25	0.007
CO6A3_HUMAN	Collagen α-3(VI) chain	41	16.53	4.428	4.43	0.000
MARE1_HUMAN	Microtubule-associated protein RP/EB family member 1	2	7.463	89.966	89.97	0.018

### TCs *versus* FBs, 5th day in cell culture

#### Identification of the differentially expressed protein

We found that in TCs, as compared to FBs, there are 39 up-regulated proteins, especially Myosin-14 (15.72-fold). We subsequently examined the FBs by comparison with TCs and found that there are 25 up-regulated proteins, especially collagen alfa 3(VI) chain (4.71-fold), secernin-1 (3.73-fold), fascin (3.25-fold) as detailed in Tables S1 and S2.

#### Bioinformatic analyses of the identified proteins

**Figure 2:**
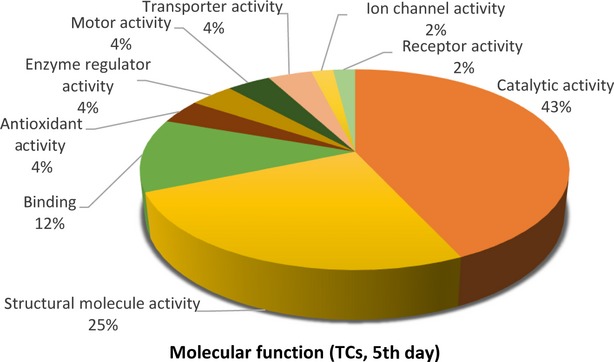
Pie chart representation of the distribution of identified proteins in TCs (cell culture, 5th day) according to their molecular functions. Categorizations were based on information provided by the online resource PANTHER classification system.

The distributions of differentially abundant proteins in putative functional categories are shown in Figures 7 according to the PANTHER. The various *molecular functions* which were found to be mapped with the participation of the identified proteins in TCs are involved mainly in catalytic activity (43%) and as structural molecule activity (25%; Fig. 2) compared with FBs in which they are chiefly involved in binding (32%), catalytic activity (28%) and as structural molecule activity (28%; Fig. 5).

**Figure 3:**
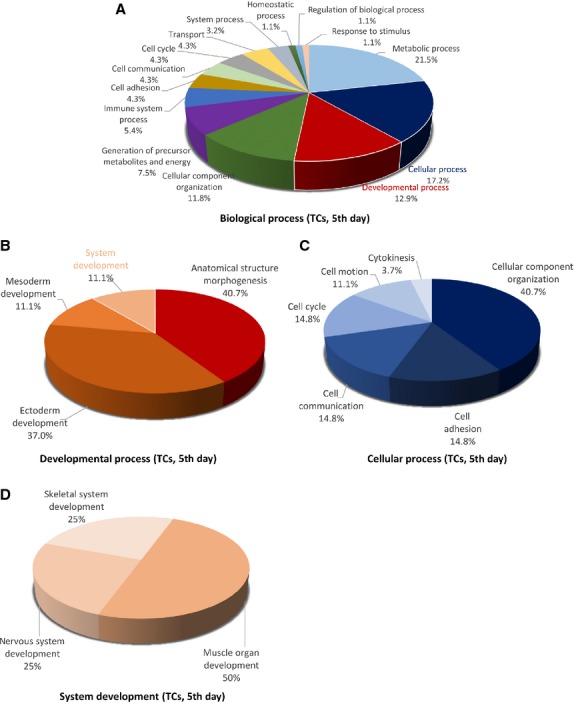
Pie chart representation of the distribution of identified proteins in TCs (cell culture, 5th day) according to their biological processes (A), cellular processes (B), developmental processes (C) and system development (D) involvement.

**Figure 4:**
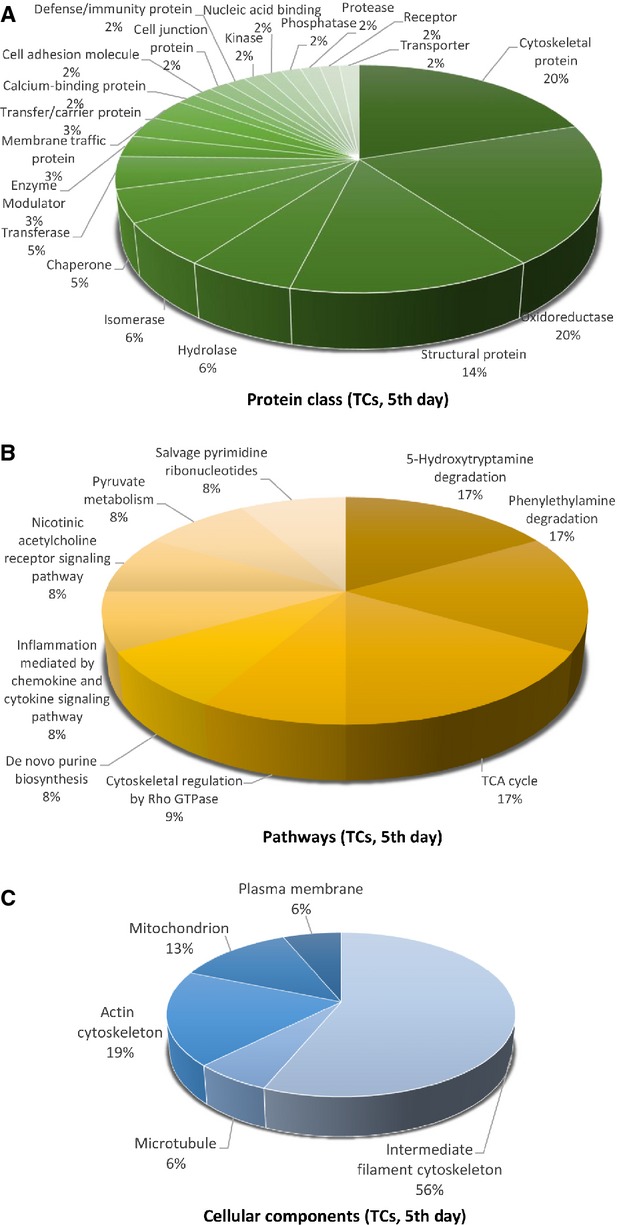
Pie chart representation of the distribution of identified proteins in TCs (cell culture, 5th day) according to their protein class (A), pathways (B) and cellular components (C) classifications.

**Figure 5:**
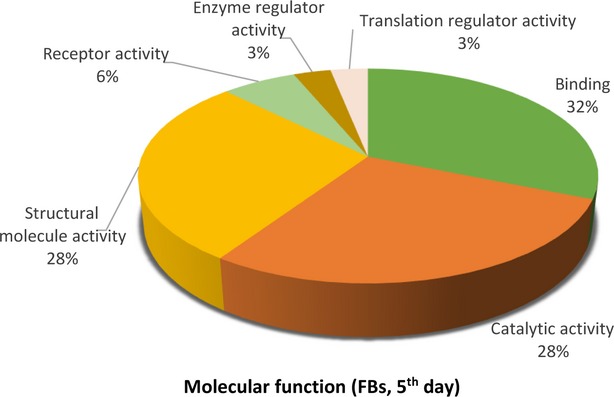
Pie chart representation of the distribution of identified proteins in FBs (cell culture, 5th day) according to their molecular functions. Categorizations were based on information provided by the online resource PANTHER classification system.

**Figure 6:**
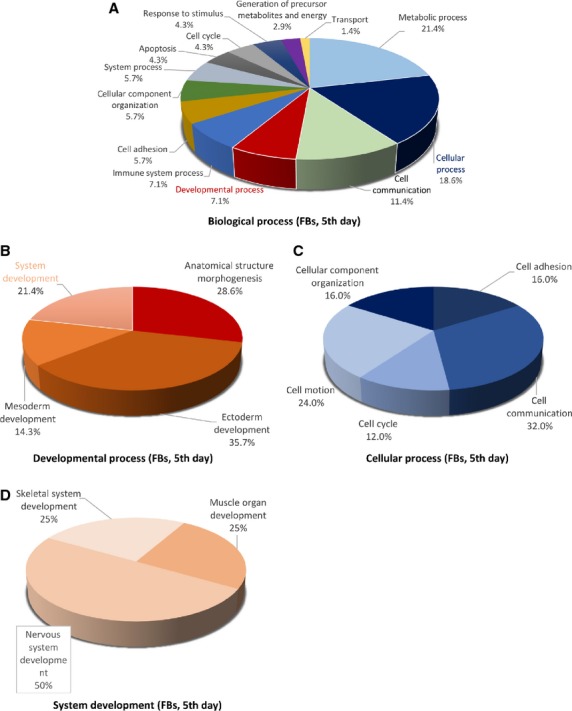
Pie chart representation of the distribution of identified proteins in FBs (cell culture, 5th day) according to their biological processes (A), cellular processes (B), developmental processes (C) and system development (D) involvement.

**Figure 7:**
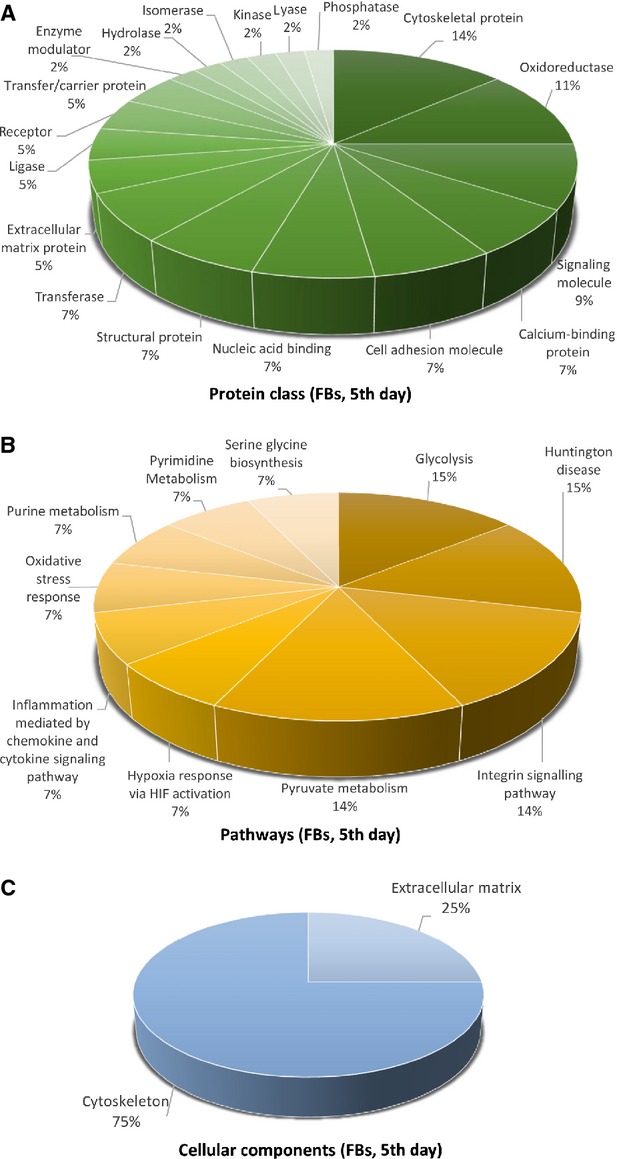
Pie chart representation of the distribution of identified proteins in FBs (cell culture, 5th day) according to their protein class (A), pathways (B) and cellular components (C) classifications.

The 39 proteins identified in TCs were assigned to following main *biological processes*: metabolic process (21.5%), cellular process (17.2%), developmental process (12.9%) and cellular component organization (11.8%), generation of precursor metabolites and energy (7.5%) immune system process (5.4%), cell communication, transport, cell adhesion, cell cycle (each 4.3%), system process (3.2%), homoeostatic process, regulation of biological process, response to stimulus (each 1.1%; Fig. 3A). The 25 proteins over expressed in FBs were assigned dominantly to metabolic process (21.4%), cellular process (18.6%), cell communication (11.4%), developmental and immune system processes (each 7.1%) and cell adhesion (5.7%; Fig. 6A).

The main *developmental processes* which engage TCs proteins are anatomical structure morphogenesis (˜41%), ectoderm development (37%), mesoderm development and system development (each ˜11%; Fig. 3B), comparative to FBs where proteins are engaged in anatomical structure morphogenesis (˜29%), ectoderm development (35.7%), mesoderm development (14.3%) and system development (21.4%) respectively (Fig. 6B).

The *cellular processes* which involve TCs proteins are cellular component organization (˜41%), cell communication, cell adhesion and cell cycle (each ˜15%; Fig. 3C), while FBs proteins are involved in cell communication (32%), cell motion (24%), cell adhesion and cellular component organization (each 16%) and cell cycle (12%; Fig. 6C).

There is a big difference regarding *system development functions* between TCs and FBs: muscle organ development 50%, nervous system and skeletal muscle development 25% each in TCs (Fig. 3D) and 50% nervous system development and skeletal muscle and muscle organ development 25% each for FBs (Fig. 6D).

The proteins in TCs are attributed to the following *protein classes*: cytoscheletal proteins (20%), oxidoreductase (20%) and structural proteins (14%; Fig. 4A), hydrolase and isomerase (each 6%), chaperone and transferase (each 5%), while in FBs proteins pertain to cytoscheletal proteins (14%), oxidoreductase (11%), signalling molecule (9%), calcium-binding protein, cell adhesion molecule, nucleic acid binding, structural proteins and transferase (each 7%; Fig. 7A).

The different highly significant *pathway* map associated with the proteins in TCs showed that they are involved in 5-HT degradation (17%), TCA cycle (17%) and phenyletylamine degradation (17%) and cytoskeletal regulation by Rho GTPase (9%) and *de novo* purine shynthesis (8%; Fig. 4B). By comparison, FBs proteome clearly depicts that they are involved in glycolysis (15%), Huntington disease (15%), integrin signalling pathway (14%), pyruvate metabolism and (14%; Fig. 7B).

The *cellular localization* of TCs proteome demonstrated proteins from intermediate filaments (56%), actin cytoscheleton (19%), mitochondria (13%), microtubule (6%) and plasma membrane (6%; Fig. 4C). In FBs, 75% of the proteins belong to the cytoskeleton and 25% are destined to the extracellular matrix (Fig. 7C).

**Figure 8:**
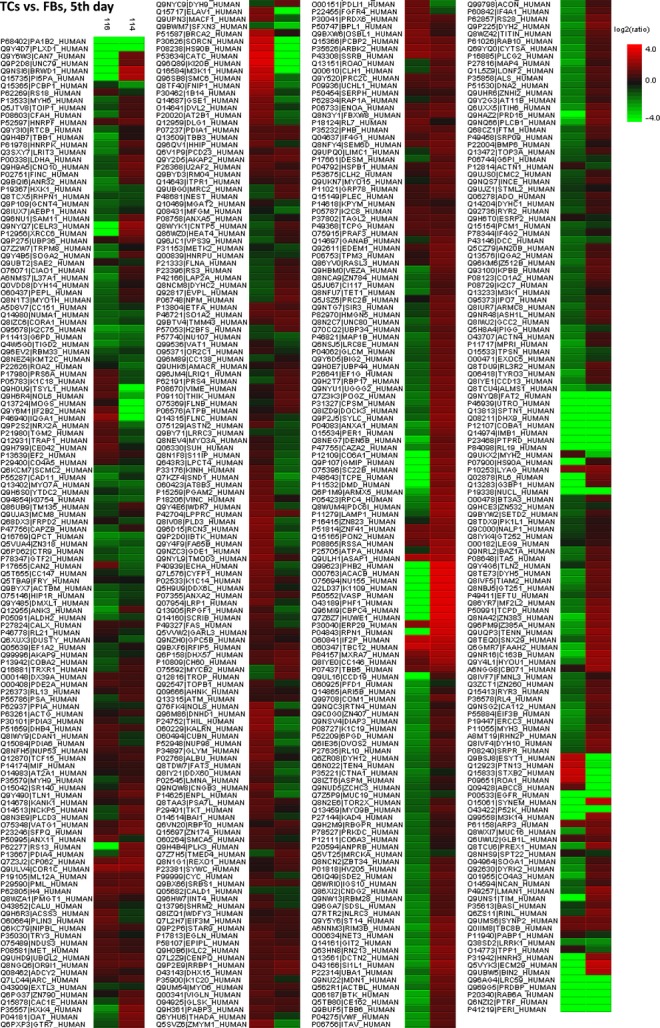
Heat map depicting significance results between TCs and FBs (cell culture, 5th day). Experimental samples are clustered on the horizontal axis and protein spots on the vertical axis. Red indicates increased and green decreased expression ratio, while black squares indicate no change in protein abundance. The colour gradient indicates the magnitude of fold change.

Figure 8 represents the heat map based on the results of protein quantification and demonstrates that proteins are differentially expressed between TCs and FBs.

### TCs *versus* FBs, 10th day in cell culture

#### Identification of the differentially expressed protein

We discovered that in TCs, as compared to FBs, there are 24 up-regulated proteins, especially superoxide dismutase (8.36-fold) and prostacyclin synthase 6A (8.12-fold). Myosin-14 remains up-regulated at 10 days (6.6-fold). In FBs, there are 40 up-regulated proteins especially Microtubule-associated protein RP/EB family member 1 (89.96-fold) and Collagen α-3(VI) chain (4.428-fold) comparative to TCs (Tables S3 and S4).

#### Bioinformatic analyses of the identified proteins

**Figure 9:**
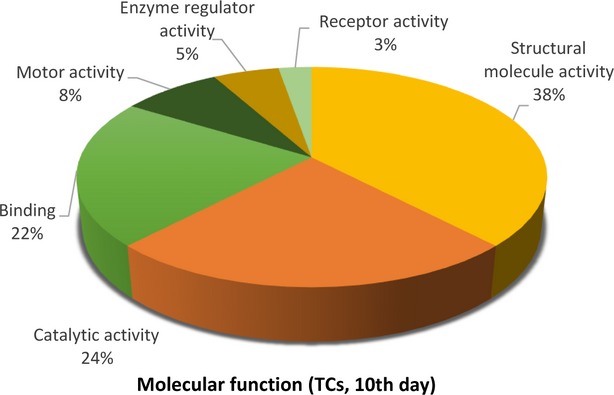
Pie chart representation of the distribution of identified proteins in TCs (cell culture, 10th day) according to their molecular functions. Categorizations were based on information provided by the online resource PANTHER classification system.

Figures9 14 show the distribution of proteins in presumed functional categories according to the PANTHER. The various *molecular functions* which were attributed of the identified proteins in TCs are involved mainly in structural molecule activity (38%), in catalytic activity (24%) and binding (22%; Fig. 9) compared with FBs where are mostly implicated in binding (36%), structural molecule activity (29%), catalytic activity (13%; Fig. 2).

**Figure 10:**
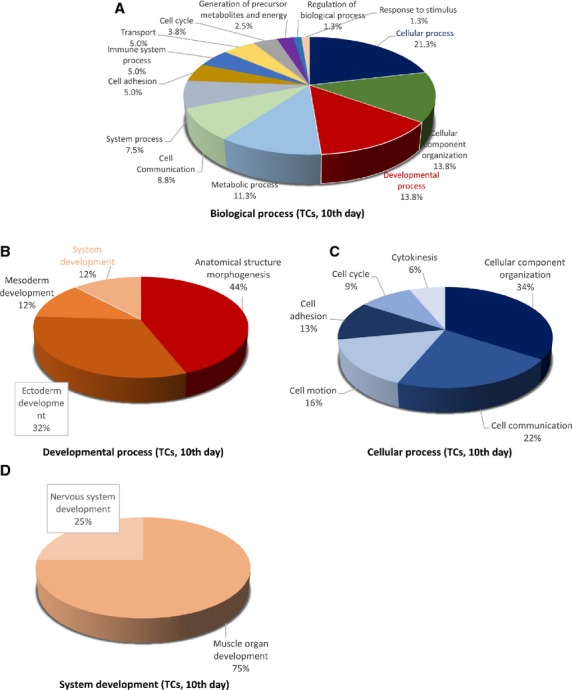
Pie chart representation of the distribution of identified proteins in TCs (cell culture, 10th day) according to their biological processes (A), cellular processes (B), developmental processes (C) and system development (D) involvement.

**Figure 11:**
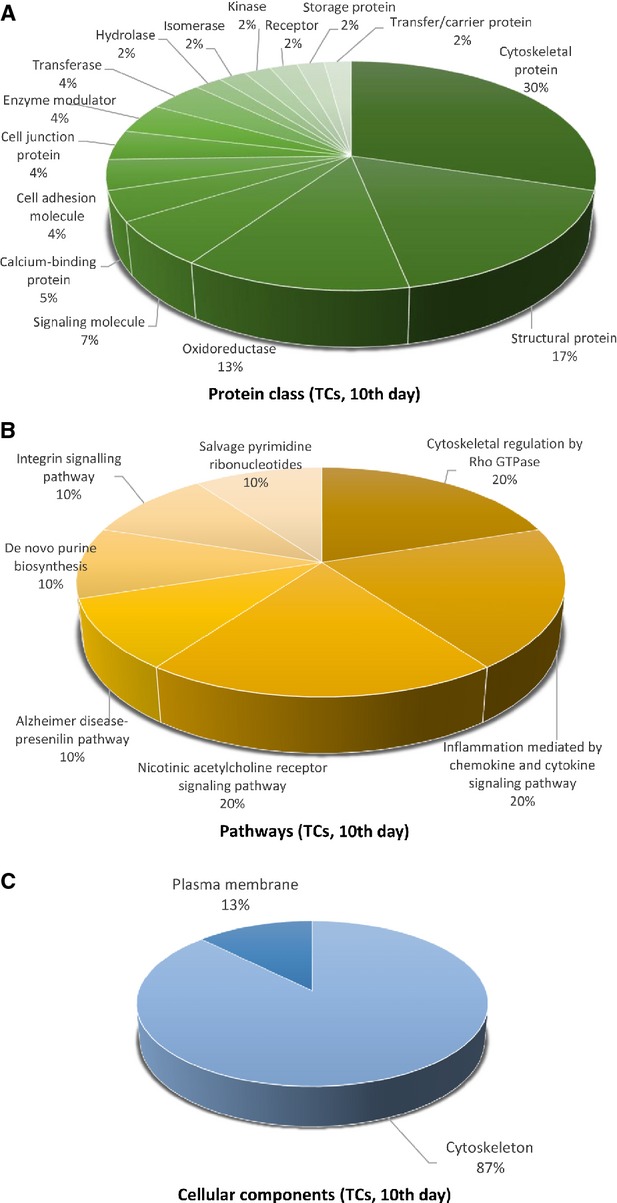
Pie chart representation of the distribution of identified proteins in TCs (cell culture, 10th day) according to their protein class (A), pathways (B, and cellular components (C) classifications.

**Figure 12:**
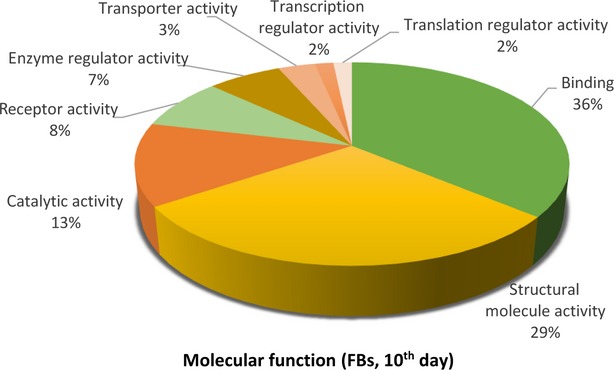
Pie chart representation of the distribution of identified proteins in FBs (cell culture, 10th day) according to their molecular functions. Categorizations were based on information provided by the online resource PANTHER classification system.

**Figure 13:**
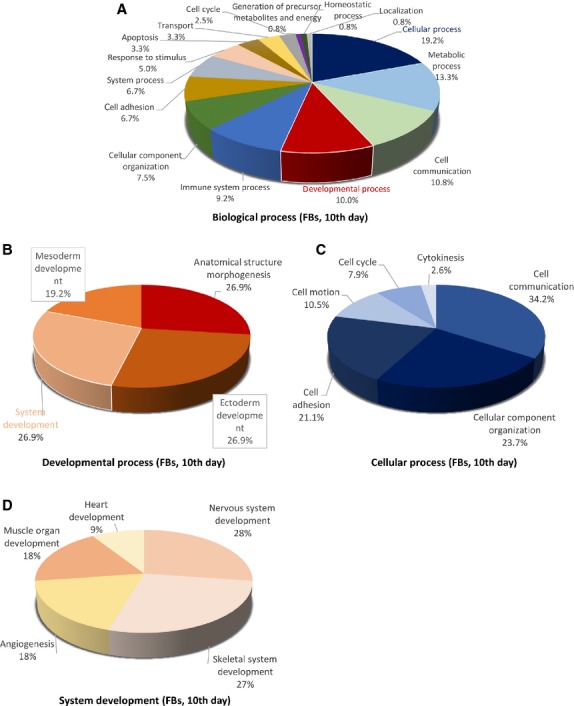
Pie chart representation of the distribution of identified proteins in FBs (cell culture, 10th day) according to their biological processes (A), cellular processes (B), developmental processes (C) and system development (D) involvement.

**Figure 14:**
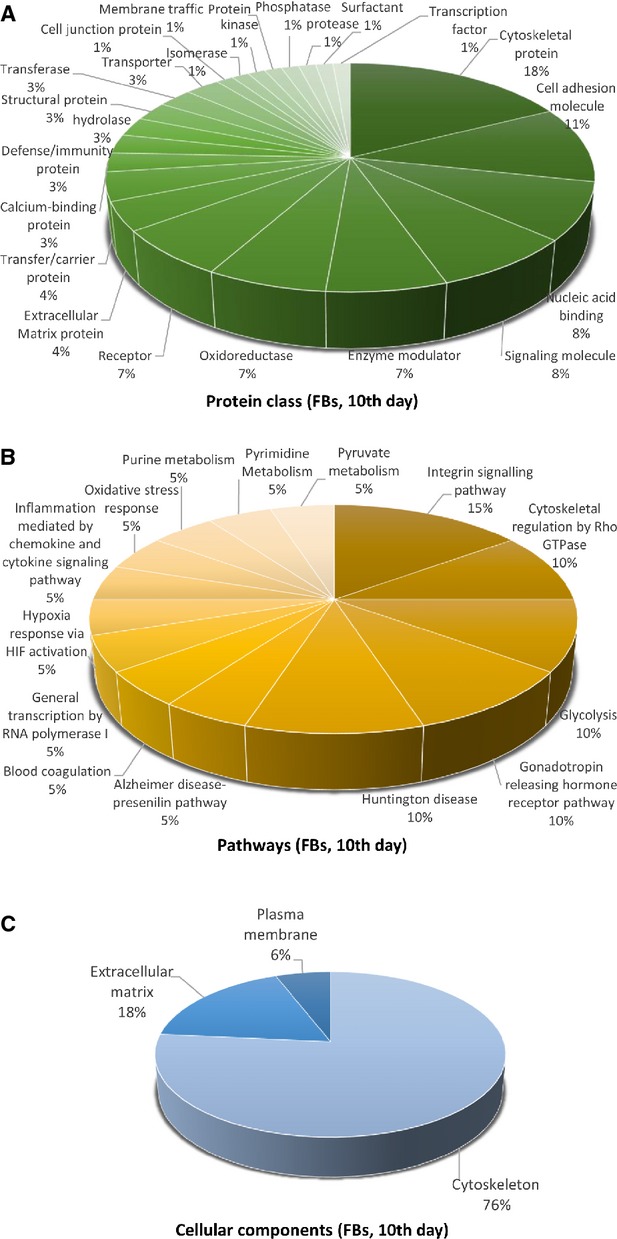
Pie chart representation of the distribution of identified proteins in FBs (cell culture, 10th day) according to their protein class (A), pathways (B) and cellular components (C) classifications.

The 39 proteins identified in TCs were assigned to following main *biological processes*: cellular process (21.3%), developmental process (13.8%), cellular component organization (13.8%) and metabolic process (11.3%), cell–cell communication (8.8%), system processes (7.5%) cell adhesion (5%), immune system process (5%), transport (5%), generation of precursor metabolites and energy (2.5%), cell cycle (3.8%), regulation of biological process, response to stimulus (each 1.3%; Fig. 10A). The 25 proteins over expressed in FBs were assigned dominantly to cellular process (19.2%), metabolic processes (13.3%), cell communication (10.8%), developmental processes (10%), immune system process (9.2%), cellular component organization (7.5%), cell adhesion (6.7%), system process (6.7%), response to stimulus (5%) apoptosis and transport 3.3% each (Fig. 13A).

The main *developmental processes* which employ TCs proteins are anatomical structure morphogenesis (44%) and ectoderm development (32%) mesoderm and system development (each 12%; Fig. 10B), comparative to FBs where anatomical structure morphogenesis, ectoderm development and system development are in equal proportion ˜27% each and mesoderm development (19.2%; Fig. 13B).

The *cellular processes* which engage TCs proteins are cellular component organization (34%), cell communication (22%), cell motion (16%) and cell adhesion (13%; Fig. 10C), while FBs proteins are involved in cell communication (34.2%), cellular component organization (˜24%), cell adhesion (˜21%), cell motion (10%; Fig. 13C).

There is a big difference regarding *system development functions* between TCs and FBs: muscle organ development 75%, nervous system development 25% in TCs (Fig. 10D) and 50% nervous system development (28%), skeletal system development (27%) muscle organ development (18%) and angigenesis (18%) and heart development (9%) for FBs (Fig. 13D). The proteins in TCs are attributed to the following *protein classes*: cytoscheletal proteins (30%), structural proteins (17%) and oxidoreductase (13%), signalling molecule (7%), calcium-binding protein (5%) and cell adhesion molecule, cell junction protein, enzyme modulator and transferase (each 4%; Fig. 11A), while in FBs proteins belong to cytoscheletal proteins (18%), cell adhesion molecule (11%), signalling molecule (8%), nucleic acid binding calcium (8%), oxidoreductase (7%), enzyme modulator (7%), receptor (7%; Fig. 14A).

The most significant *pathway* in TCs showed that they are involved mainly in nicotinic acethylcoline receptor signalling pathway (20%), inflammation mediated by chemokine and cytokine (20%) and cytoskeletal regulation by Rho GTPase (20%), *de novo* purine shynthesis (10%), integrin signalling pathway (10%), salvage pyrimidine nucleotides (10%) and Alzheimer disease-presenilin pathways (10%; Fig. 11B). By comparison, FBs proteome clearly depicts that they are involved in synthesizing the extracellular matrix components: integrin signalling pathway (15%), cytoskeletal regulation by Rho GTPase, glycolysis, gonadotropin releasing hormone pathway and Huntington disease (each 10%; Fig. 14B).

The *cellular localization* of TCs proteome demonstrated proteins from cytoscheleton (87%) and plasma membrane (13%; Fig. 11C). In FBs, 76% of the proteins belong to the cytoskeleton, 18% to the extracellular matrix and 6% to the plasma membrane (Fig. 14C).

**Figure 15:**
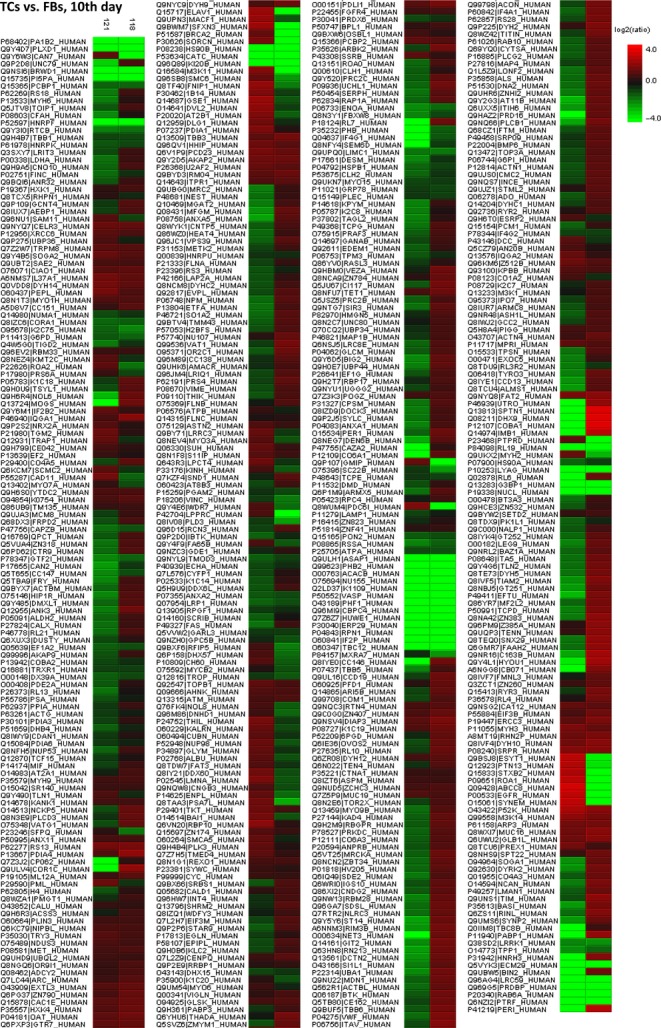
Heat map depicting significance results between TCs and FBs (cell culture, 10th day). Experimental samples are clustered on the horizontal axis and protein spots on the vertical axis. Red indicates increased and green decreased expression ratio, while black squares indicate no change in protein abundance. The colour gradient indicates the magnitude of fold change.

The heat map showing the expression of differentially expressed protein between TCs and FBs is showed in Figure 15 and demonstrated that the differences between this two cell types are still maintained in cell culture after 10 days.

**Figure 16:**
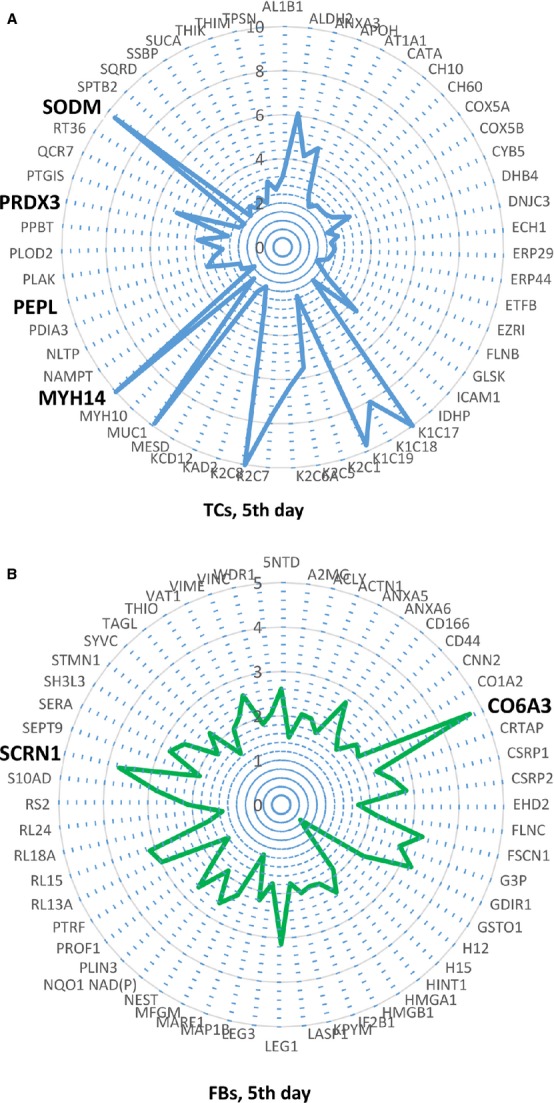
(–B) Radars of differential protein expression at 5th day in cell culture for top proteins of TCs (A) and FBs (B). For display purposes, high values of fold change was limited to 10 in A and to 5 in B, respectively. For proteins with fold change greater than 2, the corresponding fold change value was taken into account, even if lower than 2.

**Figure 17:**
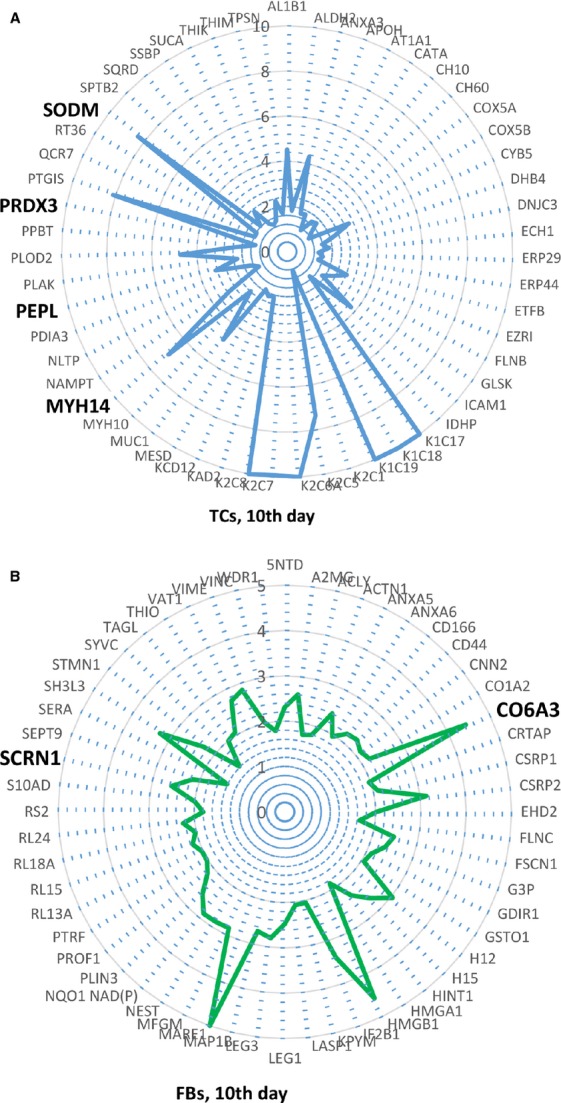
(A, B) Radars of differential protein expression at 10th day in cell culture for top proteins of TCs (A) and FBs (B). For display purposes, high values of fold change was limited to 10 in A and to 5 in B respectively. For proteins with fold change greater than 2, the corresponding fold change value was taken into account, even if lower than 2.

As presented by radar representation both cell types, TCs and FBs suffer a slight switch in phenotype between the 5th and 10th day, however Figures 16A, B and 17A, B clearly show major differences between TCs and FBs.

## Discussion

The domain of proteomics enables comparative analysis of existing proteins in different cells 49. In addition, it allows monitoring the changes in protein expression during the dynamic transformations that cells undergo during their differentiation 50. For this reason, we performed a proteomic analysis of TCs compared to FBs, at different time-points (the 5th and 10th day in primary cell culture), to verify if these cells are different from one another and if there are any changes in their differentiation (phenotype) over time. After the identification of targeted proteins, we characterized and associated them with protein families using PANTHER database and analysed them from the functional point of view.

We identified a panel of 1609 proteins from human lung TCs and FBs cell cultures samples which were quantified and the highest fold change expressed proteins between TCs and FBs were analysed and classified using the protein database. According to our results, there were top 39 ranked proteins over expressed in TCs compared to FBs at 5th day in cell culture and 24 up-regulated proteins in TCs by comparison to FBs at 10th day in cell culture and this supports the case for TCs as distinctive cells.

### Putative roles of differentially expressed proteins in TCs

In TCs, by comparison with FBs, the differentially identified proteins were mainly located in the cytoplasmic compartment and involved in cell signalling, energy and metabolic pathways, while a significant part of the FBs proteins were destined to the extracellular matrix which is in concordance with its well known function of producing extracellular matrix components (including collagen) 51.

**Myosin-14**, which was found to be up-regulated in TCs, is a conventional non-muscle myosin encoded by the MYH14 gene in human chromosome 19q13.3 52. We found that non-muscle myosin-14 seems to be involved in processes such as *sensory perception*,*intercellular signalling* and *morphogenesis*, according to the PANTHER classification system. Among previous assumptions on TCs functions it has been included that of mechanoreceptors 53, capable of detecting and translate stretch information to the nucleus 12,16. The presence of cytoskeleton proteins and especially myosin-14 in TCs confirms this hypothesis. Taking into account that myosin-14 is known to be involved in sensory perception 54, we can propose TCs as candidates for a *mechanical sensing and mechanochemical conversion task*. In addition, the presence of TC primary cilia was reported in vasculature 55 and trachea 56, functioning as mechano- or chemosensors 57, probably involved in the initiation of cellular global positioning to initiate tissue renewal after damage 58.

Telocyte proteome also revealed the presence of periplakin. **Periplakin**, a protein which in humans is encoded by the PPL gene, links cytoskeleton elements together and connects them to junctional complexes 59. Up till now, electron microscopy studies revealed that TCs establish *homocellular (TC–TC junctions) and heterocellular junctions (TC-other cell type)* 25,26. Mechanical junctions are essential to the proliferation, migration and transformation of various cell types 60,61 and therefore we might suggest that TCs are involved in these mentioned processes. Also, *intercellular signalling* is pivotal in switching cells between different fates such as growth, differentiation and motility, and it could be influenced by tensional force generation within the cytoskeleton 62,63. We suggest that TCs might participate in *mechanical sensing and mechanochemical conversion task* and also in *tissue repair/remodelling/renewal*, as shown before 38,39,64,65.

Telocytes proteomic analysis revealed the up-regulation of proteins with oxidoreductase activity, mostly located within mitocondria. It had been proved under electron microscope that TCs have calcium uptake/release units defined by a close relation between caveolae, endoplasmic reticulum and mithocondria 66, located in the podoms of telopodes 12,16,26. It is well known that mitochondria and endoplasmic reticulum establish bilateral physiological interactions responsible (among numerous functions) for modulating the calcium signalling function, process which involves redox and redoxsensitive enzymes 67. ER–mitochondria crosstalk is essential for eukaryotic cells preventing the onset of diseases by disrupted metabolism 68,69. Moreover, Haines *et al*. suggested that the intensity of oxidative stress influences cell tissue composition towards desired cell type ratio, functioning as a cell death ‘rheostat’ 70. Therefore, we consider that *TCs are involved in the maintenance of cell and tissue homoeostasis*.

Mammalian cells release extracellular vesicles produced by two mechanisms: (a) secretion from the endosomal membrane compartment after the fusion of multivesicular bodies with a plasma membrane and (b) shedding directly from plasma membrane 71. Telocytes are no exception to this, the presence of *exosomes and ectosomes* being recently reported 7,16,26. *Several proteins up-regulated in TCs were found among the top 100 vesicular proteins that are present most frequently in mammalian extracellular vesicles proteome* 72. Among them we can exemplify with proteins such as: mitochondrial thioredoxin-dependent peroxide reductase, protein disulphide-isomerase A3, myosin-14, myosin-10, filamin-B, sodium/potassium-transporting ATPase subunit α-1 and keratin, type II cytoskeletal 1. We can assume that the release of extracellular proteins contributes to the extracellular environment homoeostasis, possibly influencing stem cell niches, leading to cell differentiation which is in congruence with very recent studies pointing out that TCs could function as an extensive intercellular information transmission system 73.

## Conclusion

The present study represents the first proteomic analysis on TCs and will provide useful insights on the possible functions of these cells and involvement in lung (and not only) pathology. The data herein reported show that TCs are completely different from FBs, not only by their ultrastructural configuration [1, 23], gene profile [42], immunophenotype [74], but also from the protein expression point of view. The data presented now are supporting for our previous assumptions regarding TCs functions in tissue morphogenesis, development and repair/remodelling, extracellular environment homoeostasis and intercellular signalling influencing stem cell niche fate.

## Supporting information

**Table S1.** Summary of up-regulated TCs proteins (more than twofold) *versus* FBs 5th day. **Table S2.** Summary of up-regulated FBs proteins (more than two-fold) *versus* TCs 5th day. **Table S3.** Summary of up-regulated TCs proteins (more than two-fold) *versus* FBs 10th day. **Table S4.** Summary of up-regulated FBs proteins (more than two-fold) *versus* TCs 10th day.
