# Supplementary material for: Comparative proteomic analysis of human lung telocytes with fibroblasts
Source: J Cell Mol Med. 2014 Mar 28;18(4):568–89. doi: 10.1111/jcmm.12290 (PMC4000110; doi:10.1111/jcmm.12290)
Supplement: Supplementary file 1 — Table S1. Summary of up-regulated TCs proteins (more than twofold) versus FBs 5th day. Table S2. Summary of up-regulated FBs proteins (more than two-fold) versus TCs 5th day. Table S3. Summary of up-regulated TCs proteins (more than two-fold) versus FBs 10th day. Table S4. Summary of up-regulated FBs proteins (more than two-fold) versus TCs 10th day. [file jcmm0018-0568-sd1.docx]

Table 1

| Accession | Protein name | Peptides  (95%) | %Cov  (95) | iTRAQ ratio  FBs:TCs | Fold  enrichment in TCs | P |
| --- | --- | --- | --- | --- | --- | --- |
| MYH14_HUMAN | Myosin-14 | 18 | 7.67 | 0.064 | 15.72 | 0.025 |
| SODM_HUMAN | Superoxide dismutase [Mn], mitochondrial | 10 | 35.14 | 0.104 | 9.6 | 0.000 |
| ALDH2_HUMAN | Aldehyde dehydrogenase, mitochondrial | 5 | 10.06 | 0.164 | 6.1 | 0.000 |
| PTGIS_HUMAN | Prostacyclin synthase | 7 | 15.80 | 0.199 | 5.03 | 0.005 |
| APOH_HUMAN | Beta-2-glycoprotein 1 | 2 | 4.35 | 0.210 | 4.75 | 0.041 |
| PPBT_HUMAN | Alkaline phosphatase, tissue-nonspecific isozyme | 2 | 4.01 | 0.260 | 3.85 | 0.043 |
| PEPL_HUMAN | Periplakin | 4 | 2.85 | 0.284 | 3.52 | 0.032 |
| AL1B1_HUMAN | Aldehyde dehydrogenase X, mitochondrial | 5 | 13.93 | 0.313 | 3.2 | 0.024 |
| THIM_HUMAN | 3-ketoacyl-CoA thiolase, mitochondrial | 12 | 33.50 | 0.329 | 3.04 | 0.036 |
| KAD2_HUMAN | Adenylate kinase 2, mitochondrial | 8 | 41.42 | 0.336 | 2.97 | 0.001 |
| PLOD2_HUMAN | Procollagen-lysine,2-oxoglutarate 5-dioxygenase 2 | 15 | 22.39 | 0.367 | 2.72 | 0.000 |
| AT1A1_HUMAN | Sodium/potassium-transporting ATPase subunit alpha-1 | 17 | 18.77 | 0.375 | 2.67 | 0.000 |
| COX5B_HUMAN | Cytochrome c oxidase subunit 5B, mitochondrial | 4 | 34.11 | 0.380 | 2.63 | 0.006 |
| DNJC3_HUMAN | DnaJ homolog subfamily C member 3 | 2 | 3.77 | 0.392 | 2.55 | 0.024 |
| PRDX3_HUMAN | Thioredoxin-dependent peroxide reductase, mitochondrial | 6 | 23.83 | 0.395 | 2.53 | 0.028 |
| SUCA_HUMAN | Succinyl-CoA ligase [GDP-forming] subunit alpha, mitochondrial | 5 | 17.63 | 0.399 | 2.51 | 0.008 |
| COX5A_HUMAN | Cytochrome c oxidase subunit 5A, mitochondrial | 6 | 58.67 | 0.408 | 2.45 | 0.000 |
| CH10_HUMAN | 10 kDa heat shock protein, mitochondrial | 12 | 73.53 | 0.409 | 2.44 | 0.003 |
| CH60_HUMAN | 60 kDa heat shock protein, mitochondrial | 60 | 62.30 | 0.414 | 2.42 | 0.003 |
| SQRD_HUMAN | Sulfide:quinone oxidoreductase, mitochondrial | 9 | 19.78 | 0.421 | 2.38 | 0.000 |
| ERP29_HUMAN | Endoplasmic reticulum resident protein 29 | 7 | 28.74 | 0.422 | 2.37 | 0.001 |
| K2C1_HUMAN | Keratin, type II cytoskeletal 1 | 12 | 15.06 | 0.435 | 2.3 | 0.004 |
| ERP44_HUMAN | Endoplasmic reticulum resident protein 44 | 4 | 8.13 | 0.442 | 2.26 | 0.002 |
| CATA_HUMAN | Catalase | 5 | 12.90 | 0.460 | 2.18 | 0.007 |
| ETFB_HUMAN | Electron transfer flavoprotein subunit beta | 6 | 25.88 | 0.473 | 2.11 | 0.008 |
| SSBP_HUMAN | Single-stranded DNA-binding protein, mitochondrial | 6 | 50.68 | 0.476 | 2.1 | 0.028 |
| NLTP_HUMAN | Non-specific lipid-transfer protein | 9 | 12.43 | 0.477 | 2.1 | 0.010 |
| PDIA3_HUMAN | Protein disulfide-isomerase A3 | 45 | 52.48 | 0.480 | 2.08 | 0.019 |
| IDHP_HUMAN | Isocitrate dehydrogenase [NADP], mitochondrial | 5 | 9.29 | 0.492 | 2.03 | 0.000 |
| SPTB2_HUMAN | Spectrin beta chain, brain 1 | 43 | 19.42 | 0.493 | 2.03 | 0.000 |
| RT36_HUMAN | 28S ribosomal protein S36, mitochondrial | 2 | 30.10 | 0.494 | 2.02 | 0.036 |
| Accession | **Protein name** | **Peptides  (95%)** | **%Cov  (95)** | **iTRAQ ratio  FBs:TCs** | **Fold  enrichment**  **In FBs** | **P** |
| THIO_HUMAN | Thioredoxin | 5 | 51.43 | 2.006 | 2.01 | 0.008 |
| KPYM_HUMAN | Pyruvate kinase isozymes M1/M2 | 36 | 67.23 | 2.035 | 2.03 | 0.016 |
| CD166_HUMAN | CD166 antigen | 4 | 11.32 | 2.055 | 2.06 | 0.006 |
| WDR1_HUMAN | WD repeat-containing protein 1 | 10 | 18.15 | 2.061 | 2.06 | 0.001 |
| RS2_HUMAN | 40S ribosomal protein S2 | 10 | 31.06 | 2.088 | 2.09 | 0.000 |
| ACLY_HUMAN | ATP-citrate synthase | 11 | 11.08 | 2.089 | 2.09 | 0.000 |
| FLNC_HUMAN | Filamin-C | 73 | 32.40 | 2.160 | 2.16 | 0.000 |
| GSTO1_HUMAN | Glutathione S-transferase omega-1 | 2 | 9.54 | 2.181 | 2.18 | 0.028 |
| VAT1_HUMAN | Synaptic vesicle membrane protein VAT-1 homolog | 12 | 31.81 | 2.183 | 2.18 | 0.015 |
| CRTAP_HUMAN | Cartilage-associated protein | 2 | 3.74 | 2.273 | 2.27 | 0.049 |
| SYVC_HUMAN | Valyl-tRNA synthetase | 3 | 2.37 | 2.276 | 2.28 | 0.010 |
| VIME_HUMAN | Vimentin | 200 | 83.69 | 2.603 | 2.6 | 0.000 |
| 5NTD_HUMAN | 5'-nucleotidase | 6 | 14.81 | 2.603 | 2.6 | 0.000 |
| NEST_HUMAN | Nestin | 22 | 16.84 | 2.615 | 2.62 | 0.006 |
| PLIN3_HUMAN | Perilipin-3 | 9 | 26.50 | 2.625 | 2.63 | 0.000 |
| ANXA6_HUMAN | Annexin A6 | 21 | 31.35 | 2.714 | 2.71 | 0.005 |
| SERA_HUMAN | D-3-phosphoglycerate dehydrogenase | 4 | 8.07 | 2.773 | 2.77 | 0.017 |
| G3P_HUMAN | Glyceraldehyde-3-phosphate dehydrogenase | 42 | 64.48 | 2.819 | 2.82 | 0.001 |
| S10AD_HUMAN | Protein S100-A13 | 5 | 44.90 | 2.823 | 2.82 | 0.000 |
| RL15_HUMAN | 60S ribosomal protein L15 | 2 | 7.84 | 3.130 | 3.13 | 0.006 |
| LEG1_HUMAN | Galectin-1 | 27 | 91.11 | 3.133 | 3.13 | 0.000 |
| GDIR1_HUMAN | Rho GDP-dissociation inhibitor 1 | 4 | 18.14 | 3.241 | 3.24 | 0.001 |
| FSCN1_HUMAN | Fascin | 6 | 16.63 | 3.255 | 3.25 | 0.004 |
| SCRN1_HUMAN | Secernin-1 | 2 | 5.80 | 3.734 | 3.73 | 0.019 |
| CO6A3_HUMAN | Collagen alpha-3(VI) chain | 41 | 16.53 | 4.707 | 4.71 | 0.000 |

Table 2

| Accession | Protein name | Peptides  (95%) | %Cov  (95) | iTRAQ ratio  FBs:TCs | Fold  enrichment in TCs | P |
| --- | --- | --- | --- | --- | --- | --- |
| SODM_HUMAN | Superoxide dismutase [Mn], mitochondrial | 10 | 35.14 | 0.120 | 8.36 | 0.001 |
| PTGIS_HUMAN | Prostacyclin synthase | 7 | 15.8 | 0.123 | 8.12 | 0.001 |
| MYH14_HUMAN | Myosin-14 | 18 | 7.669 | 0.144 | 6.96 | 0.036 |
| PLOD2_HUMAN | Procollagen-lysine,2-oxoglutarate 5-dioxygenase 2 | 15 | 22.39 | 0.211 | 4.74 | 0.000 |
| ANXA3_HUMAN | Annexin A3 | 3 | 12.69 | 0.231 | 4.32 | 0.027 |
| ICAM1_HUMAN | Intercellular adhesion molecule 1 | 6 | 14.66 | 0.267 | 3.75 | 0.001 |
| NAMPT_HUMAN | Nicotinamide phosphoribosyltransferase | 3 | 5.906 | 0.325 | 3.08 | 0.005 |
| CYB5_HUMAN | Cytochrome b5 | 4 | 32.09 | 0.333 | 3 | 0.004 |
| EZRI_HUMAN | Ezrin | 19 | 31.4 | 0.352 | 2.84 | 0.002 |
| MYH10_HUMAN | Myosin-10 | 45 | 19.59 | 0.419 | 2.38 | 0.007 |
| FLNB_HUMAN | Filamin-B | 96 | 41.78 | 0.430 | 2.33 | 0.000 |
| THIM_HUMAN | 3-ketoacyl-CoA thiolase, mitochondrial | 12 | 33.5 | 0.434 | 2.3 | 0.000 |
| SQRD_HUMAN | Sulfide:quinone oxidoreductase, mitochondrial | 9 | 19.78 | 0.435 | 2.3 | 0.000 |
| PLAK_HUMAN | Junction plakoglobin | 3 | 4.564 | 0.447 | 2.24 | 0.011 |
| DHB4_HUMAN | Peroxisomal multifunctional enzyme type 2 | 11 | 25 | 0.456 | 2.19 | 0.030 |
| KAD2_HUMAN | Adenylate kinase 2, mitochondrial | 8 | 41.42 | 0.484 | 2.07 | 0.000 |
| Accession | **Protein name** | **Peptides  (95%)** | **%Cov  (95)** | **iTRAQ ratio  FBs:TCs** | **Fold  enrichment in FBs** | **P** |
| TAGL_HUMAN | Transgelin | 15 | 75.12 | 1.996 | 2 | 0.000 |
| EHD2_HUMAN | EH domain-containing protein 2 | 8 | 17.13 | 2.021 | 2.02 | 0.000 |
| RL18A_HUMAN | 60S ribosomal protein L18a | 3 | 17.61 | 2.022 | 2.02 | 0.002 |
| RL13A_HUMAN | 60S ribosomal protein L13a | 2 | 6.897 | 2.023 | 2.02 | 0.003 |
| PTRF_HUMAN | Polymerase I and transcript release factor | 17 | 42.82 | 2.024 | 2.02 | 0.036 |
| VINC_HUMAN | Vinculin | 38 | 36.95 | 2.027 | 2.03 | 0.000 |
| KPYM_HUMAN | Pyruvate kinase isozymes M1/M2 | 36 | 67.23 | 2.054 | 2.05 | 0.004 |
| GSTO1_HUMAN | Glutathione S-transferase omega-1 | 2 | 9.544 | 2.059 | 2.06 | 0.008 |
| LASP1_HUMAN | LIM and SH3 domain protein 1 | 6 | 22.61 | 2.068 | 2.07 | 0.000 |
| THIO_HUMAN | Thioredoxin | 5 | 51.43 | 2.072 | 2.07 | 0.017 |
| CSRP1_HUMAN | Cysteine and glycine-rich protein 1 | 3 | 21.76 | 2.105 | 2.11 | 0.003 |
| GDIR1_HUMAN | Rho GDP-dissociation inhibitor 1 | 4 | 18.14 | 2.121 | 2.12 | 0.024 |
| CNN2_HUMAN | Calponin-2 | 9 | 37.86 | 2.131 | 2.13 | 0.000 |
| SEPT9_HUMAN | Septin-9 | 3 | 5.119 | 2.158 | 2.16 | 0.010 |
| PROF1_HUMAN | Profilin-1 | 23 | 75.71 | 2.176 | 2.18 | 0.000 |
| CO1A2_HUMAN | Collagen alpha-2(I) chain | 9 | 7.906 | 2.202 | 2.2 | 0.012 |
| CD166_HUMAN | CD166 antigen | 4 | 11.32 | 2.203 | 2.2 | 0.009 |
| CD44_HUMAN | CD44 antigen | 7 | 9.569 | 2.251 | 2.25 | 0.012 |
| RL24_HUMAN | 60S ribosomal protein L24 | 6 | 31.21 | 2.256 | 2.26 | 0.002 |
| STMN1_HUMAN | Stathmin | 6 | 32.21 | 2.303 | 2.3 | 0.003 |
| 5NTD_HUMAN | 5'-nucleotidase | 6 | 14.81 | 2.309 | 2.31 | 0.007 |
| G3P_HUMAN | Glyceraldehyde-3-phosphate dehydrogenase | 42 | 64.48 | 2.349 | 2.35 | 0.000 |
| ANXA5_HUMAN | Annexin A5 | 30 | 54.37 | 2.409 | 2.41 | 0.003 |
| FSCN1_HUMAN | Fascin | 6 | 16.63 | 2.467 | 2.47 | 0.001 |
| LEG1_HUMAN | Galectin-1 | 27 | 91.11 | 2.472 | 2.47 | 0.000 |
| PLIN3_HUMAN | Perilipin-3 | 9 | 26.5 | 2.568 | 2.57 | 0.000 |
| A2MG_HUMAN | Alpha-2-macroglobulin | 7 | 4.342 | 2.606 | 2.61 | 0.027 |
| H15_HUMAN | Histone H1.5 | 7 | 23.45 | 2.692 | 2.69 | 0.009 |
| MAP1B_HUMAN | Microtubule-associated protein 1B | 11 | 5.146 | 2.700 | 2.7 | 0.036 |
| VAT1_HUMAN | Synaptic vesicle membrane protein VAT-1 homolog | 12 | 31.81 | 2.717 | 2.72 | 0.000 |
| LEG3_HUMAN | Galectin-3 | 10 | 30.4 | 2.800 | 2.8 | 0.000 |
| MFGM_HUMAN | Lactadherin | 6 | 17.05 | 2.844 | 2.84 | 0.019 |
| VIME_HUMAN | Vimentin | 200 | 83.69 | 2.848 | 2.85 | 0.000 |
| NEST_HUMAN | Nestin | 22 | 16.84 | 2.865 | 2.87 | 0.007 |
| NQO1_HUMAN NAD(P) | H dehydrogenase [quinone] 1 | 2 | 7.664 | 2.874 | 2.87 | 0.006 |
| H12_HUMAN | Histone H1.2 | 7 | 30.99 | 3.039 | 3.04 | 0.001 |
| CSRP2_HUMAN | Cysteine and glycine-rich protein 2 | 1 | 7.772 | 3.135 | 3.14 | 0.022 |
| SH3L3_HUMAN | SH3 domain-binding glutamic acid-rich-like protein 3 | 3 | 34.41 | 3.252 | 3.25 | 0.007 |
| CO6A3_HUMAN | Collagen alpha-3(VI) chain | 41 | 16.53 | 4.428 | 4.43 | 0.000 |
| MARE1_HUMAN | Microtubule-associated protein RP/EB family member 1 | 2 | 7.463 | 89.966 | 89.97 | 0.018 |

Table 3

| Nr. crt. | Protein name | Gene  ▲ | Molecular Function | Biological Process | Cellular Component | Protein Class | Pathway |
| --- | --- | --- | --- | --- | --- | --- | --- |
| 1 | Aldehyde dehydrogenase X, mitochondrial | AL1B1 | oxidoreductase activity | nucleobase, nucleoside, nucleotide and nucleic acid metabolic process; cellular amino acid metabolic process |  | dehydrogenase | 5-Hydroxytryptamine degredation → Aldehyde Dehydrogenase; Phenylethylamine degradation → Phenylacetaldehyde dehydrogenase |
| 2 | Aldehyde dehydrogenase, mitochondrial | ALDH2 | oxidoreductase activity | nucleobase, nucleoside, nucleotide and nucleic acid metabolic process; cellular amino acid metabolic process |  | dehydrogenase | 5-Hydroxytryptamine degredation → Aldehyde Dehydrogenase; Phenylethylamine degradation → Phenylacetaldehyde dehydrogenase |
| 3 | Beta-2-glycoprotein 1 | APOH | serine-type peptidase activity; metallopeptidase activity; receptor activity; lipid transporter activity | complement activation; signal transduction; cell-cell adhesion; proteolysis; signal transduction; cell-cell adhesion; blood coagulation |  | apolipoprotein; receptor; metalloprotease; serine protease; metalloprotease; serine protease; complement component; cell adhesion molecule |  |
| 4 | Sodium/potassium-transporting ATPase subunit alpha-1 | AT1A1 | hydrolase activity; cation transmembrane transporter activity; ion channel activity | cation transport; cellular calcium ion homeostasis |  | cation transporter; ion channel; hydrolase |  |
| 5 | Catalase | CATA | oxidoreductase activity; peroxidase activity | immune system process; respiratory electron transport chain; oxygen and reactive oxygen species metabolic process |  | peroxidase |  |
| 6 | 10 kDa heat shock protein, mitochondrial | CH10 |  | protein metabolic process |  | chaperonin |  |
| 7 | 60 kDa heat shock protein, mitochondrial | CH60 |  | protein folding; protein complex assembly |  | chaperonin |  |
| 8 | Cytochrome c oxidase subunit 5A, mitochondrial | COX5A | oxidoreductase activity | oxidative phosphorylation; respiratory electron transport chain |  | oxidase |  |
| 9 | Cytochrome c oxidase subunit 5B, mitochondrial | COX5B | oxidoreductase activity | oxidative phosphorylation; respiratory electron transport chain | mitochondrion; organelle | oxidase |  |
| 10 | DnaJ homolog subfamily C member 3 | DNJC3 | protein disulfide isomerase activity; protein binding; kinase inhibitor activity; kinase regulator activity | intracellular protein transport; exocytosis; cell cycle; synaptic transmission; protein folding; protein modification process; cell cycle; synaptic transmission |  | membrane traffic protein; isomerase; kinase inhibitor; chaperone |  |
| 11 | Endoplasmic reticulum resident protein 29 | ERP29 |  | intracellular protein transport; exocytosis |  | membrane traffic protein |  |
| 12 | Endoplasmic reticulum resident protein 44 | ERP44 | protein disulfide isomerase activity | protein modification process |  | isomerase |  |
| 13 | Electron transfer flavoprotein subunit beta | ETFB | oxidoreductase activity | respiratory electron transport chain |  | hydroxylase |  |
| 14 | Isocitrate dehydrogenase [NADP], mitochondrial | IDHP | oxidoreductase activity | tricarboxylic acid cycle; carbohydrate metabolic process |  | dehydrogenase | TCA cycle → Isocitrate Dehydrogenase |
| 15 | Keratin, type II cytoskeletal 1 | K2C1 | structural constituent of cytoskeleton | cellular component morphogenesis; ectoderm development | intermediate filament cytoskeleton | structural protein; intermediate filament |  |
| 16 | Adenylate kinase 2, mitochondrial | KAD2 | nucleotide kinase activity | purine base metabolic process; pyrimidine base metabolic process |  | nucleotide kinase; nucleotide kinase | Salvage pyrimidine ribonucleotides → Cytidylate kinase; De novo purine biosynthesis → Adenylate kinase |
| 17 | Myosin-14 | MYH14 | motor activity; structural constituent of cytoskeleton; protein binding; small GTPase regulator activity | muscle contraction; sensory perception of sound; sensory perception; intracellular protein transport; vesicle-mediated transport; mitosis; intracellular signaling cascade; cytokinesis; cell motion; mitosis; cellular component morphogenesis; mesoderm development; muscle organ development | actin cytoskeleton; cell junction | G-protein modulator; actin binding motor protein; cell junction protein | Inflammation mediated by chemokine and cytokine signaling pathway → Myosin; Nicotinic acetylcholine receptor signaling pathway → Myosin; Cytoskeletal regulation by Rho GTPase → Myosin light chain |
| 18 | Non-specific lipid-transfer protein | NLTP | oxidoreductase activity; acetyltransferase activity | protein amino acid acetylation |  | acetyltransferase; transfer/carrier protein; dehydrogenase; reductase |  |
| 19 | Protein disulfide-isomerase A3 | PDIA3 | protein disulfide isomerase activity | protein modification process |  | isomerase |  |
| 20 | Periplakin | PEPL | structural constituent of cytoskeleton; intermediate filament binding | cell adhesion; cell adhesion; cellular component morphogenesis; ectoderm development | intermediate filament cytoskeleton | intermediate filament binding protein |  |
| 21 | Procollagen-lysine,2-oxoglutarate 5-dioxygenase 2 | PLOD2 | oxidoreductase activity | cell adhesion; protein metabolic process; cell adhesion |  | oxygenase |  |
| 22 | Alkaline phosphatase, tissue-nonspecific isozyme | PPBT |  | mesoderm development; skeletal system development |  | phosphatase; phosphatase |  |
| 23 | Thioredoxin-dependent peroxide reductase, mitochondrial | PRDX3 | oxidoreductase activity; peroxidase activity | immune system process; oxygen and reactive oxygen species metabolic process |  | peroxidase |  |
| 24 | Prostacyclin synthase | PTGIS | oxidoreductase activity; isomerase activity | blood circulation; respiratory electron transport chain; fatty acid biosynthetic process; regulation of vasoconstriction |  | oxidoreductase; isomerase |  |
| 25 | 28S ribosomal protein S36, mitochondrial | RT36 |  |  |  |  |  |
| 26 | Superoxide dismutase [Mn], mitochondrial | SODM | oxidoreductase activity | immune system process; oxygen and reactive oxygen species metabolic process |  | oxidoreductase |  |
| 27 | Spectrin beta chain, brain 1 | SPTB2 | structural constituent of cytoskeleton; calcium ion binding; actin binding | muscle contraction; neurological system process; cell cycle; nitric oxide mediated signal transduction; neuromuscular synaptic transmission; cell adhesion; cell motion; cell cycle; cellular component morphogenesis; ectoderm development; mesoderm development; nervous system development; muscle organ development | actin cytoskeleton | structural protein; non-motor actin binding protein; calcium-binding protein |  |
| 28 | Sulfide:quinone oxidoreductase, mitochondrial | SQRD | oxidoreductase activity | sulfur metabolic process |  | reductase |  |
| 29 | Single-stranded DNA-binding protein, mitochondrial | SSBP | single-stranded DNA binding | cell cycle; DNA replication; cell cycle |  | DNA binding protein |  |
| 30 | Succinyl-CoA ligase [GDP-forming] subunit alpha, mitochondrial | SUCA | catalytic activity | tricarboxylic acid cycle; carbohydrate metabolic process |  |  | Pyruvate metabolism → Citrate Lyase; TCA cycle → Succinyl CoA Synthetase |
| 31 | 3-ketoacyl-CoA thiolase, mitochondrial | THIM | acetyltransferase activity | protein amino acid acetylation |  | acetyltransferase |  |

Table 4

| Nr.crt. | Protein name | Gene  ▲ | Molecular Function | Biological Process | Cellular Component | Protein Class | Pathway |
| --- | --- | --- | --- | --- | --- | --- | --- |
| 1 | 5'-nucleotidase | 5NTD | phosphoric diester hydrolase activity; nucleotide phosphatase activity; nucleotide phosphatase activity | nucleobase, nucleoside, nucleotide and nucleic acid metabolic process |  | nucleotide phosphatase; nucleotide phosphatase; phosphodiesterase | Pyrimidine Metabolism → 5'-Nucleotidase; Purine metabolism → 5'-Nucleotidase |
| 2 | ATP-citrate synthase | ACLY | transferase activity; lyase activity; ligase activity | tricarboxylic acid cycle; coenzyme metabolic process; carbohydrate metabolic process; lipid metabolic process |  | transferase; lyase; ligase | Pyruvate metabolism → Citrate Lyase |
| 3 | Annexin A6 | ANXA6 | calcium ion binding; calcium-dependent phospholipid binding | synaptic vesicle exocytosis; intracellular protein transport; exocytosis; calcium-mediated signaling; fatty acid metabolic process; cell motion |  | transfer/carrier protein; annexin |  |
| 4 | CD166 antigen | CD166 | receptor activity | signal transduction; cell-cell adhesion; ectoderm development; nervous system development |  | receptor; immunoglobulin superfamily cell adhesion molecule |  |
| 5 | Collagen alpha-3(VI) chain | CO6A3 | receptor activity; extracellular matrix structural constituent | immune system process; sensory perception of sound; sensory perception; signal transduction; cell-matrix adhesion; cell-cell adhesion; cell motion; cellular component morphogenesis; ectoderm development; mesoderm development; skeletal system development; blood coagulation | extracellular matrix | receptor; extracellular matrix structural protein; cell adhesion molecule | Integrin signalling pathway → Collagen; Inflammation mediated by chemokine and cytokine signaling pathway → ExtraCellular matrix protein |
| 6 | Cartilage-associated protein | CRTAP |  |  | extracellular matrix | extracellular matrix protein |  |
| 7 | Filamin-C | FLNC | structural constituent of cytoskeleton; calcium ion binding; actin binding | muscle contraction; neurological system process; cell cycle; nitric oxide mediated signal transduction; neuromuscular synaptic transmission; cell adhesion; cell motion; cellular component morphogenesis; ectoderm development; mesoderm development; nervous system development; muscle organ development | actin cytoskeleton | structural protein; non-motor actin binding protein; calcium-binding protein | Integrin signalling pathway → Filamin |
| 8 | Fascin | FSCN1 | structural constituent of cytoskeleton; actin binding | cell motion | actin cytoskeleton | non-motor actin binding protein |  |
| 9 | Glyceraldehyde-3-phosphate dehydrogenase | G3P | oxidoreductase activity | glycolysis |  | dehydrogenase | Glycolysis → Glyceraldehyde 3-phosphate dehydrogenase; Huntington disease → Glyceraldehyde-3-phosphate dehydrogenase |
| 10 | Rho GDP-dissociation inhibitor 1 | GDIR1 | receptor binding; small GTPase regulator activity | intracellular signaling cascade |  | signaling molecule; G-protein modulator |  |
| 11 | Glutathione S-transferase omega-1 | GSTO1 | oxidoreductase activity; transferase activity; racemase and epimerase activity; structural constituent of cytoskeleton; translation factor activity, nucleic acid binding; receptor binding; translation elongation factor activity | immune system process; intracellular signaling cascade; oxygen and reactive oxygen species metabolic process; translation; response to toxin | cytoskeleton | transferase; signaling molecule; reductase; translation elongation factor; epimerase/racemase; cytoskeletal protein |  |
| 12 | Pyruvate kinase isozymes M1/M2 | KPYM | carbohydrate kinase activity | glycolysis |  | carbohydrate kinase; carbohydrate kinase | Glycolysis → Pyruvate kinase; Pyruvate metabolism → Pyruvate Kinase |
| 13 | Galectin-1 | LEG1 | receptor binding | immune system process; induction of apoptosis; cell adhesion; cell adhesion |  | signaling molecule; cell adhesion molecule |  |
| 14 | Nestin | NEST | structural constituent of cytoskeleton | cellular component morphogenesis; ectoderm development | intermediate filament cytoskeleton | structural protein; intermediate filament |  |
| 15 | Perilipin-3 | PLIN3 |  | lipid metabolic process |  | transfer/carrier protein |  |
| 16 | 60S ribosomal protein L15 | RL15 | structural constituent of ribosome; nucleic acid binding | translation |  | ribosomal protein |  |
| 17 | 40S ribosomal protein S2 | RS2 | structural constituent of ribosome; nucleic acid binding | translation |  | ribosomal protein |  |
| 18 | Protein S100-A13 | S10AD | calcium ion binding; receptor binding; calmodulin binding | macrophage activation; cell cycle; intracellular signaling cascade; DNA replication; cell motion; cell cycle |  | signaling molecule; calmodulin |  |
| 19 | Secernin-1 | SCRN1 |  | proteolysis |  |  |  |
| 20 | D-3-phosphoglycerate dehydrogenase | SERA | oxidoreductase activity | carbohydrate metabolic process; cellular amino acid biosynthetic process |  | dehydrogenase | Serine glycine biosynthesis → Phosphoglycerate dehydrogenase |
| 21 | Valyl-tRNA synthetase | SYVC | aminoacyl-tRNA ligase activity | tRNA aminoacylation for protein translation |  | aminoacyl-tRNA synthetase |  |
| 22 | Thioredoxin | THIO | oxidoreductase activity | immune system process; respiratory electron transport chain; apoptosis; meiosis; apoptosis; sulfur metabolic process; response to stress |  | oxidoreductase | Hypoxia response via HIF activation → thioredoxin; Oxidative stress response → thioredoxin |
| 23 | Synaptic vesicle membrane protein VAT-1 homolog | VAT1 | oxidoreductase activity | apoptosis; carbohydrate metabolic process |  | dehydrogenase; reductase | Huntington disease → Tumor protein p53 inducible protein 3 |
| 24 | Vimentin | VIME | structural constituent of cytoskeleton | cellular component morphogenesis; ectoderm development | intermediate filament cytoskeleton | structural protein; intermediate filament |  |
| 25 | WD repeat-containing protein 1 | WDR1 | structural constituent of cytoskeleton; actin binding | sensory perception of sound; sensory perception; cell motion | actin cytoskeleton | non-motor actin binding protein |  |

Table 5

| Nr.crt. | Protein name | Gene  ▲ | Molecular Function | Biological Process | Cellular Component | Protein Class | Pathway |
| --- | --- | --- | --- | --- | --- | --- | --- |
| 1 | Annexin A3 | ANXA3 | calcium ion binding; calcium-dependent phospholipid binding | synaptic vesicle exocytosis; intracellular protein transport; exocytosis; calcium-mediated signaling; fatty acid metabolic process; cell motion; calcium-mediated signaling |  | transfer/carrier protein; annexin |  |
| 2 | Cytochrome b5 | CYB5 | oxidoreductase activity | respiratory electron transport chain; steroid metabolic process |  | oxidase |  |
| 3 | Peroxisomal multifunctional enzyme type 2 | DHB4 | oxidoreductase activity | visual perception; sensory perception; cellular amino acid biosynthetic process; steroid metabolic process |  | dehydrogenase; reductase |  |
| 4 | Ezrin | EZRI | structural constituent of cytoskeleton | cellular component morphogenesis | actin cytoskeleton | actin family cytoskeletal protein |  |
| 5 | Filamin-B | FLNB | structural constituent of cytoskeleton; calcium ion binding; actin binding | muscle contraction; neurological system process; cell cycle; nitric oxide mediated signal transduction; neuromuscular synaptic transmission; cell adhesion; cell motion; cellular component morphogenesis; ectoderm development; mesoderm development; nervous system development; muscle organ development | actin cytoskeleton | structural protein; non-motor actin binding protein; calcium-binding protein | Integrin signalling pathway → Filamin |
| 6 | Intercellular adhesion molecule 1 | ICAM1 | receptor activity; receptor binding | B cell mediated immunity; signal transduction; cell-cell adhesion; response to stimulus |  | signaling molecule; receptor; immunoglobulin superfamily cell adhesion molecule |  |
| 7 | Adenylate kinase 2, mitochondrial | KAD2 | nucleotide kinase activity | purine base metabolic process; pyrimidine base metabolic process |  | nucleotide kinase; nucleotide kinase | Salvage pyrimidine ribonucleotides → Cytidylate kinase; De novo purine biosynthesis → Adenylate kinase |
| 8 | Myosin-10 | MYH10 | motor activity; structural constituent of cytoskeleton; protein binding; small GTPase regulator activity | muscle contraction; sensory perception of sound; sensory perception; intracellular protein transport; vesicle-mediated transport; mitosis; intracellular signaling cascade; cytokinesis; cell motion; cellular component morphogenesis; mesoderm development; muscle organ development | actin cytoskeleton; cell junction | G-protein modulator; actin binding motor protein; cell junction protein | Inflammation mediated by chemokine and cytokine signaling pathway → Myosin; Nicotinic acetylcholine receptor signaling pathway → Myosin; Cytoskeletal regulation by Rho GTPase → Myosin light chain |
| 9 | Myosin-14 | MYH14 | motor activity; structural constituent of cytoskeleton; protein binding; small GTPase regulator activity | muscle contraction; sensory perception of sound; sensory perception; intracellular protein transport; vesicle-mediated transport; mitosis; intracellular signaling cascade; cytokinesis; cell motion; cellular component morphogenesis; mesoderm development; muscle organ development | actin cytoskeleton; cell junction | G-protein modulator; actin binding motor protein; cell junction protein | Inflammation mediated by chemokine and cytokine signaling pathway → Myosin; Nicotinic acetylcholine receptor signaling pathway → Myosin; Cytoskeletal regulation by Rho GTPase → Myosin light chain |
| 10 | Nicotinamide phosphoribosyltransferase | NAMPT | cytokine activity | immune system process; signal transduction; cell-cell signaling |  | cytokine |  |
| 11 | Junction plakoglobin | PLAK | structural constituent of cytoskeleton; receptor binding | intracellular protein transport; signal transduction; cell-cell adhesion | cytoskeleton | storage protein; signaling molecule; cytoskeletal protein; cell adhesion molecule | Alzheimer disease-presenilin pathway → gamma-catenin |
| 12 | Procollagen-lysine,2-oxoglutarate 5-dioxygenase 2 | PLOD2 | oxidoreductase activity | cell adhesion; protein metabolic process; cell adhesion |  | oxygenase |  |
| 13 | Prostacyclin synthase | PTGIS | oxidoreductase activity; isomerase activity | blood circulation; respiratory electron transport chain; fatty acid biosynthetic process; regulation of vasoconstriction |  | oxidoreductase; isomerase |  |
| 14 | Superoxide dismutase [Mn], mitochondrial | SODM | oxidoreductase activity | immune system process; oxygen and reactive oxygen species metabolic process |  | oxidoreductase |  |
| 15 | Sulfide:quinone oxidoreductase, mitochondrial | SQRD | oxidoreductase activity | sulfur metabolic process |  | reductase |  |
| 16 | 3-ketoacyl-CoA thiolase, mitochondrial | THIM | acetyltransferase activity | protein amino acid acetylation |  | acetyltransferase |  |

Table 6

| Nr.crt. | Protein name | Gene  ▲ | Molecular Function | Biological Process | Cellular Component | Protein Class | Pathway |
| --- | --- | --- | --- | --- | --- | --- | --- |
| 1 | 5'-nucleotidase | 5NTD | phosphoric diester hydrolase activity; nucleotide phosphatase activity; nucleotide phosphatase activity | nucleobase, nucleoside, nucleotide and nucleic acid metabolic process |  | nucleotide phosphatase; nucleotide phosphatase; phosphodiesterase | Pyrimidine Metabolism → 5'-Nucleotidase; Purine metabolism → 5'-Nucleotidase |
| 2 | Alpha-2-macroglobulin | A2MG | cytokine activity; serine-type endopeptidase inhibitor activity | complement activation; signal transduction; cell-cell signaling; proteolysis; response to stimulus |  | cytokine; serine protease inhibitor; complement component | Blood coagulation → alpha-2-macroglobulin |
| 3 | Annexin A5 | ANXA5 | calcium ion binding; calcium-dependent phospholipid binding | synaptic vesicle exocytosis; intracellular protein transport; exocytosis; calcium-mediated signaling; fatty acid metabolic process; cell motion |  | transfer/carrier protein; annexin | Gonadotropin releasing hormone receptor pathway → Annexin A5 |
| 4 | CD166 antigen | CD166 | receptor activity | signal transduction; cell-cell adhesion; ectoderm development; nervous system development |  | receptor; immunoglobulin superfamily cell adhesion molecule |  |
| 5 | CD44 antigen | CD44 | receptor activity | immune system process; cell communication; cell adhesion | plasma membrane | receptor; cell junction protein; cell adhesion molecule | Alzheimer disease-presenilin pathway → CD44 transmembrane fragment; Alzheimer disease-presenilin pathway → CD44 C-terminal fragment; Alzheimer disease-presenilin pathway → CD44 intracellular fragment; Alzheimer disease-presenilin pathway → Cell surface glycoprotein 44; Alzheimer disease-presenilin pathway → CD44 N-terminal fragment |
| 6 | Calponin-2 | CNN2 | structural constituent of cytoskeleton; actin binding | muscle contraction | actin cytoskeleton | non-motor actin binding protein |  |
| 7 | Collagen alpha-2(I) chain | CO1A2 | receptor activity; extracellular matrix structural constituent; transmembrane transporter activity | macrophage activation; blood circulation; intracellular protein transport; receptor-mediated endocytosis; signal transduction; cell-cell adhesion; cellular component morphogenesis; ectoderm development; mesoderm development; skeletal system development; angiogenesis; regulation of liquid surface tension; defense response to bacterium; asymmetric protein localization | extracellular matrix | transporter; surfactant; receptor; extracellular matrix structural protein; antibacterial response protein; cell adhesion molecule | Integrin signalling pathway → Collagen |
| 8 | Collagen alpha-3(VI) chain | CO6A3 | receptor activity; extracellular matrix structural constituent | immune system process; sensory perception of sound; sensory perception; signal transduction; cell-matrix adhesion; cell-cell adhesion; cell motion; cellular component morphogenesis; ectoderm development; mesoderm development; skeletal system development; blood coagulation | extracellular matrix | receptor; extracellular matrix structural protein; cell adhesion molecule | Integrin signalling pathway → Collagen; Inflammation mediated by chemokine and cytokine signaling pathway → ExtraCellular matrix protein |
| 9 | Cysteine and glycine-rich protein 1 | CSRP1 | structural constituent of cytoskeleton | immune system process; mesoderm development; muscle organ development | actin cytoskeleton | actin family cytoskeletal protein |  |
| 10 | Cysteine and glycine-rich protein 2 | CSRP2 | structural constituent of cytoskeleton | immune system process; mesoderm development; muscle organ development | actin cytoskeleton | actin family cytoskeletal protein |  |
| 11 | EH domain-containing protein 2 | EHD2 | calcium ion binding; protein binding; small GTPase regulator activity | neurotransmitter secretion; intracellular protein transport; endocytosis; synaptic transmission; synaptic transmission |  | membrane traffic protein; G-protein modulator; calcium-binding protein |  |
| 12 | Fascin | FSCN1 | structural constituent of cytoskeleton; actin binding | cell motion | actin cytoskeleton | non-motor actin binding protein |  |
| 13 | Glyceraldehyde-3-phosphate dehydrogenase | G3P | oxidoreductase activity | glycolysis |  | dehydrogenase | Glycolysis → Glyceraldehyde 3-phosphate dehydrogenase; Huntington disease → Glyceraldehyde-3-phosphate dehydrogenase; |
| 14 | Rho GDP-dissociation inhibitor 1 | GDIR1 | receptor binding; small GTPase regulator activity | intracellular signaling cascade |  | signaling molecule; G-protein modulator |  |
| 15 | Glutathione S-transferase omega-1 | GSTO1 | oxidoreductase activity; transferase activity; racemase and epimerase activity; structural constituent of cytoskeleton; translation factor activity, nucleic acid binding; receptor binding; translation elongation factor activity | immune system process; intracellular signaling cascade; oxygen and reactive oxygen species metabolic process; translation; intracellular signaling cascade; response to toxin | cytoskeleton | transferase; signaling molecule; reductase; translation elongation factor; epimerase/racemase; cytoskeletal protein |  |
| 16 | Histone H1.2 | H12 | DNA binding | nucleobase, nucleoside, nucleotide and nucleic acid metabolic process; establishment or maintenance of chromatin architecture |  | histone |  |
| 17 | Histone H1.5 | H15 | DNA binding | nucleobase, nucleoside, nucleotide and nucleic acid metabolic process; establishment or maintenance of chromatin architecture |  | histone |  |
| 18 | Pyruvate kinase isozymes M1/M2 | KPYM | carbohydrate kinase activity | glycolysis |  | carbohydrate kinase; carbohydrate kinase | Glycolysis → Pyruvate kinase; Pyruvate metabolism → Pyruvate Kinase |
| 19 | LIM and SH3 domain protein 1 | LASP1 | structural constituent of cytoskeleton; actin binding | muscle contraction | actin cytoskeleton | non-motor actin binding protein |  |
| 20 | Galectin-1 | LEG1 | receptor binding | immune system process; induction of apoptosis; cell adhesion; cell adhesion |  | signaling molecule; cell adhesion molecule |  |
| 21 | Galectin-3 | LEG3 | receptor binding | immune system process; induction of apoptosis; cell adhesion; cell adhesion |  | signaling molecule; cell adhesion molecule |  |
| 22 | Microtubule-associated protein 1B | MAP1B | structural constituent of cytoskeleton; microtubule binding | cellular component morphogenesis | microtubule | non-motor microtubule binding protein |  |
| 23 | Microtubule-associated protein RP/EB family member 1 | MARE1 | structural constituent of cytoskeleton; microtubule binding | cell cycle; cell cycle; cellular component morphogenesis | microtubule | non-motor microtubule binding protein |  |
| 24 | Lactadherin | MFGM | oxidoreductase activity; serine-type peptidase activity; metallopeptidase activity; receptor activity; lipid transporter activity; transmembrane transporter activity; receptor binding; enzyme regulator activity | immune system process; visual perception; sensory perception; intracellular protein transport; endocytosis; vitamin transport; cell surface receptor linked signal transduction; synaptic transmission; cell-cell adhesion; proteolysis; ectoderm development; mesoderm development; skeletal system development; angiogenesis; nervous system development; heart development; blood coagulation | extracellular matrix | transporter; apolipoprotein; membrane-bound signaling molecule; receptor; metalloprotease; serine protease; oxidase; metalloprotease; serine protease; extracellular matrix protein; enzyme modulator; cell adhesion molecule |  |
| 25 | Nestin | NEST | structural constituent of cytoskeleton | cellular component morphogenesis; ectoderm development | intermediate filament cytoskeleton | structural protein; intermediate filament |  |
| 26 | H dehydrogenase [quinone] 1 | NQO1 |  |  |  |  |  |
| 27 | Perilipin-3 | PLIN3 |  | lipid metabolic process |  | transfer/carrier protein |  |
| 28 | Profilin-1 | PROF1 |  |  |  |  | Cytoskeletal regulation by Rho GTPase → Profilin |
| 29 | Polymerase I and transcript release factor | PTRF | transcription factor activity | termination of RNA polymerase II transcription; rRNA metabolic process |  | transcription factor | General transcription by RNA polymerase I → PTRF |
| 30 | 60S ribosomal protein L13a | RL13A | structural constituent of ribosome; nucleic acid binding | translation |  | ribosomal protein |  |
| 31 | 60S ribosomal protein L18a | RL18A | structural constituent of ribosome; nucleic acid binding | translation |  | ribosomal protein |  |
| 32 | 60S ribosomal protein L24 | RL24 | structural constituent of ribosome; nucleic acid binding | translation |  | ribosomal protein |  |
| 33 | Septin-9 | SEPT9 | GTPase activity; structural constituent of cytoskeleton; protein binding | mitosis; cytokinesis; mitosis | cytoskeleton | small GTPase; cytoskeletal protein |  |
| 34 | SH3 domain-binding glutamic acid-rich-like protein 3 | SH3L3 |  |  |  |  |  |
| 35 | Stathmin | STMN1 |  | intracellular signaling cascade; ectoderm development; nervous system development |  |  | Cytoskeletal regulation by Rho GTPase → Stathmin |
| 36 | Transgelin | TAGL | structural constituent of cytoskeleton; actin binding | muscle contraction | actin cytoskeleton | non-motor actin binding protein |  |
| 37 | Thioredoxin | THIO | oxidoreductase activity | immune system process; respiratory electron transport chain; apoptosis; meiosis; apoptosis; sulfur metabolic process; response to stress |  | oxidoreductase | Hypoxia response via HIF activation → thioredoxin; Oxidative stress response → thioredoxin |
| 38 | Synaptic vesicle membrane protein VAT-1 homolog | VAT1 | oxidoreductase activity | apoptosis; carbohydrate metabolic process |  | dehydrogenase; reductase | Huntington disease → Tumor protein p53 inducible protein 3 |
| 39 | Vimentin | VIME | structural constituent of cytoskeleton | cellular component morphogenesis; ectoderm development | intermediate filament cytoskeleton | structural protein; intermediate filament |  |
| 40 | Vinculin | VINC | structural constituent of cytoskeleton; actin binding | intracellular signaling cascade; cell adhesion; cell motion; cellular component morphogenesis | actin cytoskeleton | non-motor actin binding protein; cell adhesion molecule | Gonadotropin releasing hormone receptor pathway → Vinculin; Integrin signalling pathway → Vinculin |
